# MEF2A Is the Trigger of Resveratrol Exerting Protection on Vascular Endothelial Cell

**DOI:** 10.3389/fcvm.2021.775392

**Published:** 2022-01-03

**Authors:** Benrong Liu, Lihua Pang, Yang Ji, Lei Fang, Chao Wei Tian, Jing Chen, Changnong Chen, Yun Zhong, Wen-Chao Ou, Yujuan Xiong, Shi Ming Liu

**Affiliations:** ^1^Guangdong Key Laboratory of Vascular Diseases, State Key Laboratory of Respiratory Disease, Guangzhou Institute of Cardiovascular Disease, The Second Affiliated Hospital, Guangzhou Medical University, Guangzhou, China; ^2^Department of Emergency, The Second Affiliated Hospital, Guangzhou Medical University, Guangzhou, China; ^3^Department of General Practice, The Second Affiliated Hospital, Guangzhou Medical University, Guangzhou, China; ^4^Department of Laboratory Medicine, Panyu Hospital of Chinese Medicine, Guangzhou University of Chinese Medicine, Guangzhou, China

**Keywords:** resveratrol, vascular endothelial cell, HUVEC, Sirtuin1, myocyte enhancer factor 2A

## Abstract

Both resveratrol and myocyte enhancer factor 2A (MEF2A) may protect vascular endothelial cell (VEC) through activating the expression of SIRT1. However, the relationship between resveratrol and MEF2A is unclear. We aimed to investigate the deeper mechanism of resveratrol in protecting vascular endothelial cells and whether MEF2A plays a key role in the protective function of resveratrol. Human umbilical vein endothelial cell (HUVEC) was used for *in vitro* study, and small interfere RNA was used for silencing MEF2A. Silencing MEF2A in the vascular endothelium (VE) of ApoE^−/−^ mice was performed by tail injection with adeno associated virus expressing si-mef2a-shRNA. The results showed that treatment of HUVEC with resveratrol significantly up-regulated MEF2A, and prevented H_2_O_2_-induced but not siRNA-induced down-regulation of MEF2A. Under various experimental conditions, the expression of SIRT1 changed with the level of MEF2A. Resveratrol could rescue from cell apoptosis, reduction of cell proliferation and viability induced by H_2_O_2_, but could not prevent against that caused by silencing MEF2A with siRNA. Silencing MEF2A in VE of apoE^−/−^ mice decreased the expression of SIRT1, increased the plasma LDL-c, and abrogated the function of resveratrol on reducing triglyceride. Impaired integrity of VE and aggravated atherosclerotic lesion were observed in MEF2A silenced mice through immunofluorescence and oil red O staining, respectively. In conclusion, resveratrol enhances MEF2A expression, and the upregulation of MEF2A is required for the endothelial protective benefits of resveratrol *in vitro* via activating SIRT1. Our work has also explored the *in vivo* relevance of this signaling pathway in experimental models of atherosclerosis and lipid dysregulation, setting the stage for more comprehensive phenotyping *in vivo* and further defining the molecular mechanisms.

## Introduction

Resveratrol is a polyphenol compound that can be obtained from several dietary sources, such as grapes, apples, blueberries, plums, raspberries and peanuts, and has a variety of health-promoting effects (1). Evidences from a large number of basic scientific studies and more than 240 clinical studies support the beneficial effects of resveratrol against chronic diseases such as cardiovascular disease, diabetes, hypertension, Alzheimer's disease, liver disease, kidney disease and cancer (2, 3). Among these diseases, aging is the most common risk factor, which is usually related to the accumulation of reactive oxygen species (ROS), the increase of inflammatory lesions, the abnormality of cell proliferation and the altered angiogenesis (4–6). In the cardiovascular system, aging is surely one of the most important determinants of different diseases (4, 5). Resveratrol has long been considered as an anti-aging compound and its cardiovascular protective effect was mainly attributed to its function of antioxidant, anti-inflammatory, anti-proliferation, vascular regulation and promotion of autophagy (1, 4, 7). The monolayer, flat and slightly longer vascular endothelial cells closely arranged in the inner wall of blood vessels secrete key regulatory factors that regulate blood pressure and vascular tension. Vascular endothelial dysfunction is the early event of cardiovascular disease. A large number of studies have confirmed that resveratrol is a strong protector of vascular endothelium, and its main molecular mechanisms include increasing the production of nitric oxide (NO) in vascular endothelium by up-regulating the expression and activity of NO synthase (eNOS), down-regulating the synthesis of endothelin-1 to reduce vasoconstriction and blood pressure (1, 8), promoting mitochondrial biosynthesis and reducing the production of mitochondrial superoxides (9), preventing arterial aging by reducing the activity of PRR-ACE-AngII axis and activating ACE2-Ang-(1–7)-ATR2-MasR axis (10), reducing vascular endothelial oxidative stress by up-regulating the expression of Sirtuin1 (SIRT1), and delaying vascular endothelial cell senescence (11).

SIRT1 is a member of nicotinamide adenine dinucleotide **(**NAD+) dependent histone deacetylase class III family, and is a necessary factor in delaying cell senescence and prolonging biological life span (11), and plays an important role in protecting vascular endothelium (12–14). In vascular endothelial cells, the expression of SIRT1 may be regulated by myocyte enhancer factor 2A (MEF2A) (15). MEF2A is a member of the MEF2 family and belongs to the MADS-box superfamily. MEF2A is a very important transcriptional regulatory factor, which is necessary for cell differentiation, cell proliferation, cell survival, morphogenesis and other life processes (16, 17). The important role of MEF2A in neural differentiation, maturation and synapsis has been supported by a great deal of evidence (18–20), but its role in cardiovascular disease is still controversial. In 2003, Wang et al. (21) revealed that the 21bp deletion in exon 11 of MEF2A was co-isolated from the patient with premature coronary heart disease in a general pedigree, and MEF2A has become the first identified autosomal dominant genetic variation in the risk of coronary heart disease (named ADCAD1). However, studies in the following decades have shown that 21 bp deletion in exon 11 of MEF2A is rare in the population. The genetic variation of MEF2A gene does not explain the risk of coronary heart disease in most people (22). In recent years, the research on the function of MEF2A has rekindled the hope of MEF2A in the field of cardiovascular disease. Lu et al. (23) revealed that MEF2A/C/D is a key regulator of vascular homeostasis by studying the phenotype and transcriptome after specific deletion of MEF2A/C/D from mouse endothelium. Medrano et al. (24) reported that MEF2A precisely regulates gene expression in adult atria and ventricles, and Zhou et al. (25) found that interference of MEF2A expression in apoE knockout mice can promote atherosclerotic lesions. The results of a study in renal vascular endothelium suggest that MEF2A can regulate vascular endothelial cell migration, tube formation and anti-apoptosis (26). In our previous studies, we found that inhibiting the expression of MEF2A in human coronary artery endothelial cells significantly changed the expression profile of genes related to proliferation and inflammation, induced cell senescence and significantly down-regulated the expression of SIRT1 in human coronary artery endothelial cells (15, 27). Overexpression of MEF2A in vascular endothelial cells can inhibit the down-regulation of SIRT1 induced by hydrogen peroxide (H_2_O_2_), thus inhibits cell senescence induced by H_2_O_2_. The regulation of SIRT1 by MEF2A may be caused by directly activating the expression of PI3K (15).

In vascular endothelial cells (VEC), MEF2A is considered to be the key transcriptional activator upstream of SIRT1, and resveratrol can effectively stimulate the expression of SIRT1, so does resveratrol up-regulate the expression of SIRT1 and protect VEC depending on stimulating the expression of MEF2A? In this study, we revealed that resveratrol up-regulated SIRT1 expression, functioned anti-apoptosis and anti-atherosclerotic lesions depending on up-regulating MEF2A expression.

## Materials and Methods

### Cell Culture and Reagents

The primary human umbilical vein endothelial cell (HUVEC) was purchased from Procell Life Science and Technology Co., Ltd (CL-0122) and cultured in EBM-2 medium with 3% fetal bovine serum (Gibco) and growth factors (Gibco), and incubated at 37°C in a humidified incubator with 5% CO_2_. Resveratrol was purchased from SigmaAldrich, dissolved in DMSO for cell treatment, and added to 0.5% sodium carboxymethyl cellulose solution to form a suspension for intragastric administration to mice.

### Treatment of HUVEC With H_2_O_2_

HUVEC were seeded in 48-well plates or 96-well plates and were cultured overnight. After replacing the culture medium with fresh medium, a gradient concentration of H_2_O_2_ was added and incubated for 1, 2 and 4 h, respectively. After incubating the cells with H_2_O_2_, immediately replaced the medium with fresh medium and continued to culture according to the standard conditions.

### Treatment of HUVEC With Resveratrol

10 mM of storage solution of resveratrol was prepared by dissolving resveratrol with DMSO. A gradient concentration of resveratrol solution was added to HUVECs and incubated for 12, 24 and 48 h, respectively. After incubating the cells with resveratrol, replaced the medium with fresh medium and continued to culture for further experiments.

### Transfection of Small Interfere RNA (siRNA)

Si-RNA was transfected into HUVEC using Lipofectamine RNAI MAX (Invitrogen) transfection kit, and all operations were conducted according to the manufacturer's instructions. Briefly: before transfection, medium was replaced with the fresh complete medium, siRNA and the transfection reagents were respectively diluted with serum-free medium (Opti-MEM). The diluted siRNA and the transfection reagents were mixed sufficiently and incubated for 10 min at room temperature, then all of the mixture were transferred to the target wells of the culture plates, and the final concentration of 50 nM si-RNA was used. Then, the culture plates were gently shaken and placed in a moist incubator at 37°C with 5% carbon dioxide for culturing 6–8 h. After that, culture medium was replaced with fresh EBM-2 complete medium and the cells were cultured for 48–72 h followed by performing the subsequent experiments.

### Detection of Cell Viability

Cell Counting Kit-8 (CCK8) was used to detect cell viability according to the manufacturer's instructions, briefly: The cell culture medium was replaced with fresh serum free EBM-2 medium, 10 μl CCK8 solution was added to wells containing 100 μl medium and gently mixed, then placed in a cell incubator containing 5% CO_2_ at 37°C for 3–4 h. Optical density value at 450 nm was measured on a spectrophotometer.

### Detection of Cell Proliferation Ability

The cell proliferation ability was detected by 5-Ethynyl-2′-deoxyuridine (EdU) cell proliferation detection kit (R11053.4, Ribobio, China) according to the manufacturer's instructions, and the cells staining red were highly proliferative cells.

### Detection of Cellular Mitochondrial Membrane Potential

JC-1 staining kit (M8650,Solarbio,China) was used to detect the changes of mitochondrial membrane potential according to the manufacturer's instructions. Briefly, the medium was discarded and the cells were washed twice with PBS (pH 7.2), appropriate amount of JC-1 working solution was added and incubated in the cell incubator for 20 min, then the supernatant was removed and washed twice with 1 × JC-1 staining solution and appropriate amount of cell culture medium was added to ensure the whole bottom area to be covered, which was observed under inverted fluorescence microscope and the pictures were taken for further analysis.

### Detection of Cell Apoptosis With Flow Cytometry

The eBioscience Annexin V-FITC Apoptosis Detection Kit (BMS500FI-300, Thermo Fisher Scientific, USA) was used to detect cell apoptosis according to the manufacturer's instruction. Briefly, the cells were collected and washed once with PBS (pH 7.2), then were resuspended with 500 μl stain buffer (FBS) and centrifuged at room temperature and 1,000 rpm for 5 min. The supernatant was discarded. The cells were washed once with PBS (pH 7.2), resuspended with 200 μl binding buffer and 5 μl of Annexin V labeled with Fluorescein Isothiocyanate (FITC) was added, gently mixed and incubated in dark (room temperature) for 15 min, then 10 μl of PI solution was added followed by detecting the percentage of living cells, dead cells and apoptotic cells on a flow cytometer.

### Feeding of Mice

All animal experiments carried out in this study were reviewed and approved by the Experimental Animal Ethics Committee of the Second Affiliated Hospital of Guangzhou Medical University (Approval Number: A2019−013). The male wild-type C57BL/6J mice (WTM) and the male ApoE^−/−^ mice (ApoE^−/−^M), aged from 4 to 6 weeks, were purchased from Guangdong Experimental Animal Center. The normal feed and high-fat feed (HF60, 112252) were also purchased from Guangdong Experimental Animal Center. WTM were randomly divided into two groups (*n* = 10 per each group). ApoE^−/−^M were randomly divided into 4 groups (*n* = 10 per each group). Except the mice in one group of WTM were fed with normal diet, the others were fed with high-fat diet. All mice grew in an environment with 60–70% humidity and 23 ± 2°C room temperature, under 12 h of light and 12 h of darkness alternately.

### Design of the Short Hairpin RNA (shRNA) Expression Vector for Inhibition of Mef2a in Mouse and Preparation of Adeno-Associated Virus (AAV)

The reference sequence of mouse mef2a gene (accession No.: NM_001033713) was used as the interference target to design the shRNA AAV expression vector. The mature siRNA sequence was 5′-GGGCAGUUAUCUCAGGGUU-3′. DNA sequences expressing shRNA was placed under the control of the promoter of ICAM2 to construct an endothelial cell-specific expression vector. The packaging, purification and titer determination of mouse mef2a shRNA AAV1-type adeno-associated virus were performed by Shandong Weizhen Biotechnology Co., Ltd. Finally, mef2a-shRNA-AAV1 [pAV-ICAM2-GFP-mir30-shRNA (MEF2a)] was obtained with a titer of 4.46×10^13^ Vg /ml. The pAV-ICAM2-GFP-mir30-shRNA (NC) inserted with meaningless sequence was used and packaged as negative control (NC-shRNA-AAV1), and the titer of the NC-shRNA-AAV1 was 5.14 × 10^13^ vg/ml.

### Infection of Mice With AAV1

The AAV1 (mef2a-shRNA-AAV1 and NC-shRNA-AAV1) was respectively diluted to 1 × 10^13^ vg/ml with 0.9% sterile saline and 100 μl of this diluted AAV1 solution was injected through the tail vein for each apoE^−/−^ mouse. The infected mice were fed with high-fat diet for about 90 days.

### Intragastric Administration of Resveratrol to Mice

The resveratrol suspension was prepared by dissolving resveratrol in 0.5% sodium carboxymethyl cellulose (SCC) solution. Gastric administration of the prepared resveratrol suspension was performed once a day according to 30 mg/kg body weight per day by using gastric perfusion needle and was sustained for 90 days, while the control group was given the same dose of 0.5% SCC solution.

### Sampling From Mice

The mice were anesthetized by inhaling 2% isoflurane and adequate depth of anesthesia was monitored using toe reflex. Under deep anesthesia, the mice were sacrificed by cervical dislocation, and then blood samples were collected immediately from eyes through removing the eyeballs, followed by tissue sampling. Thoracic aorta, abdominal aorta, aortic valve and heart of the mouse were excised for using in the following experiments.

### Oil Red O Staining

The frozen sections were prepared from the fresh aortic valve. The sections and the whole aorta were fixed in 4% paraformaldehyde and dried for minutes, then the dry tissues were soaked with oil red staining solution for 8–10 min (avoiding light). The vessels or slides were taken out and immersed in 60% isopropanol solution for 3 s, then were transferred to a new 60% isopropanol solution, and were rinsed with pure water twice for 10 s each. The vessels or slides were taken out to soak in hematoxylin for 3–5 min, then were washed 3 times with 60% ethanol solution for, respectively, 5, 10 and 30 s, then were washed 2 times with pure water for 10 s each, soaked in the blue solution for 1 s, rinsed twice with pure water for, respectively, 5 and 10 s, and were sealed with glycerin gelatin. Finally, the prepared slides or vessels were observed under the microscope and images were photographed.

### Immunofluorescence

The tissue specimens were fixed in 4% paraformaldehyde for 24 h, further dehydrated with gradient concentration of alcohol, then was subjected to permeation with xylene and prepared the paraffin-embedded sections. The tissue sections were immersed in antigen-retrieval buffer (pH 8.0), then were transferred in 3% hydrogen peroxide solution and incubated at room temperature for 30 min. The sections were washed with PBS, blocked with BSA, and incubated with the primary antibody at 4°C overnight. After being washed with PBS, the sections were incubated with the secondary antibody at room temperature for 50 min. After being washed with PBS, the sections were dried and incubated with TSA-FITC solution for 10 min, then were rinsed with TBST for 3 times. After eliminating obvious liquid, the slides were incubated with spontaneous fluorescence quenching reagent for 5 min, washed under flowing water for 10 min. Then, the slides were incubated with DAPI solution at room temperature for 10 min, and washed 3 times with PBS (pH 7.4), mounted with anti-fade mounting medium. Then the slides were detected and imaged with fluorescent microscopy. The primary antibodies of MEF2A (cat.gb11965) and CD31 (cat.gb11063-2) were purchased from Wuhan Servicebio Technology Co., Ltd (Wuhan, China).

### RNA Isolation and Real-Time Fluorescence Quantitative PCR

Total RNA was prepared from cells by using Trizol according to the manufacturer's instruction. The quality of RNA was detected by gel electrophoresis, and the concentration was calculated with the absorbance value detected on a spectrophotometer (Bio Tek, Epoch) at a wave length of 260 nm. Reverse transcription and synthesis of the cDNA from the total RNA was performed by using the FastKing gDNA dispelling RT superMix kit (Tiangen Bio, Beijing, China). Quantitative PCR was performed using RealUniversal color premix kit (SYBR Green) according to the manufacturer's instructions. Beta-actin (ACTB) was used as the internal control.

### Immunobloting

Cells were washed with precooled PBS (pH 7.2) and then lysed in RIPA buffer containing proteinase inhibitor. The protein content was quantified with a BCA protein assay kit (Thermo, USA). Equal amount of protein from different samples was loaded into the SDS-PAGE gel for immunobloting. The primary antibodies against MEF2A (cat.9736s, CST, USA), SIRT1 (cat.9475, CST, USA), Bcl-2 (cat.15071, CST, USA), Bax (cat.5023s, CST, USA), Caspase-3 (cat.9662s, CST, USA), Cleaved Caspase-3 (cat.9661s, CST, USA), β-actin (cat.4970s, CST, USA) and GAPDH (cat.5174s, CST, USA) were used for probing specific proteins. Beta-actin and GAPDH were used as the internal control.

### Statistical Analysis

Detection of variance homogeneity and normal distribution of data in each Group was performed by using exploratory analysis in descriptive analysis in SPSS19 software. The significance of the difference between the two groups with uniform variance was examined by Student's *t* test or two-way ANOVA with Bonferroni posterior tests. For the significance test of difference between two groups with uneven variance, the Mann-Whitney U test was used. Individual values were presented in dot plots and the mean ± standard deviation (SD) was shown with it. The difference was considered significant when a *P* value < 0.05.

## Results

### Resveratrol Promotes the Expression of MEF2A

**Figure 1:**
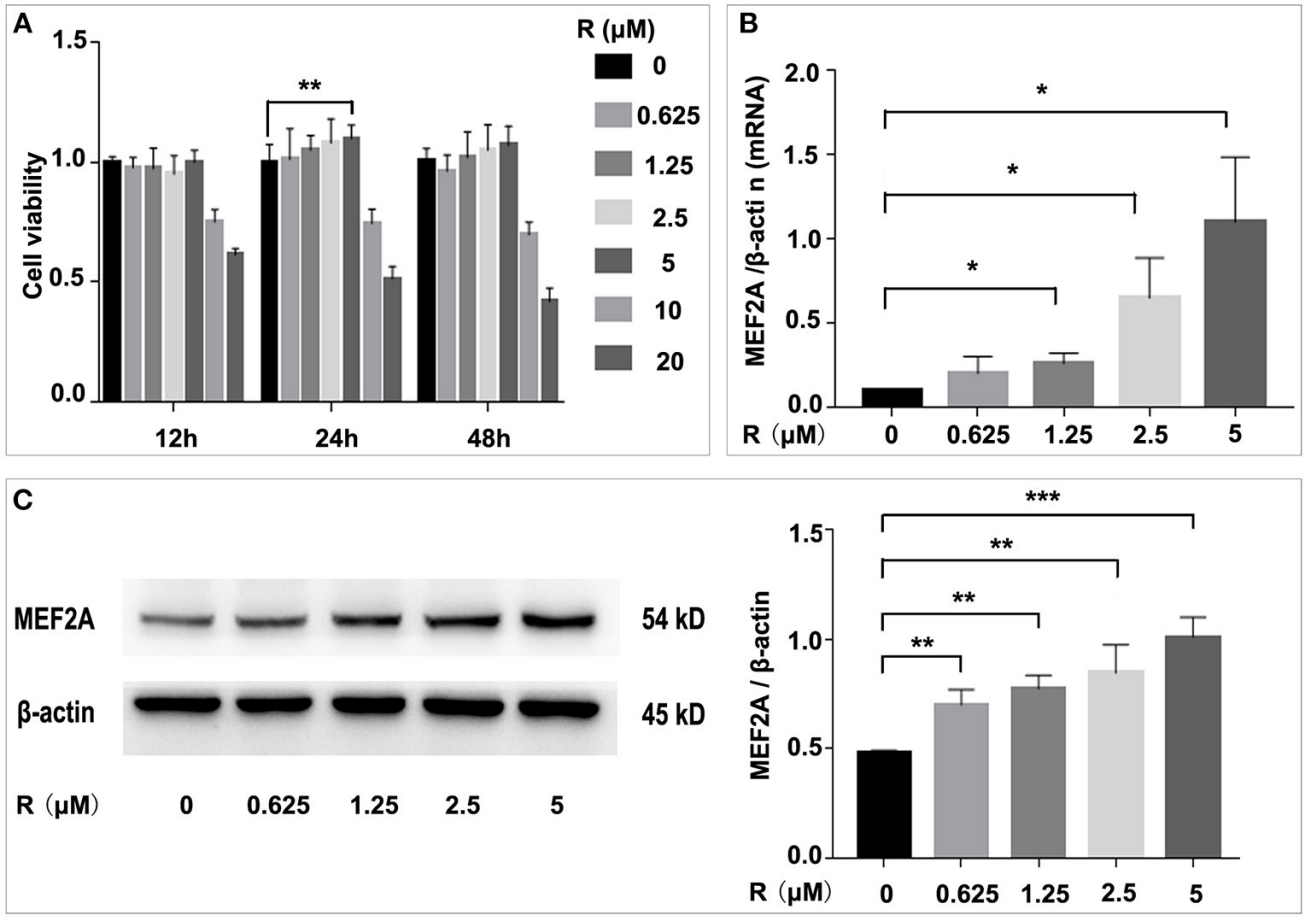
Influence of resveratrol on cell viability and the expression of MEF2A. **(A)** Impact of treatment of HUVEC with gradient concentration of resveratrol for various time on cell viability. **(B)** The mRNA level of MEF2A in HUVEC treated with gradient concentration of resveratrol for 24 h. β-actin was used as the internal control to normalize the MEF2A mRNA level. **(C)** Immunoblots of MEF2A. β-actin was used as a loading control. Quantification of band intensity was performed via image-J and values were normalized to beta-actin. All experiments were performed independently for three times. Mean ± standard deviation (SD) is showed as bar plot. The statistical significance is analyzed by the unpaired Student's two-tailed *t*-test. ns, no significance; *P < 0.05; **P < 0.01; ***P < 0.001 between groups indicated.

To determine the effect of resveratrol on HUVEC and on MEF2A expression, we treated HUVEC with resveratrol at a gradient concentration for 12, 24 and 48 h, respectively. The results showed that the HUVEC cell viability was slightly increased after treated with resveratrol of 0.625, 1.25, 2.5 and 5 μM for 24 h, and the resveratrol of 5 μM could significantly increase cell viability, while the resveratrol of 10 and 20 μM could significantly decrease cell viability. It was suggested that 5 μM of resveratrol had a protective effect on HUVEC, while 10 μM or higher resveratrol had toxic side effects on cells (Figure 1A). Thus, resveratrol of 5 μM was used in subsequent experiments. The expression of MEF2A was detected by qPCR and Western blot, and the results showed that resveratrol promoted the expression of MEF2A at mRNA and protein level in a concentration-dependent manner (Figures 1B,C). The protein level of MEF2A was doubled when HUVECs were treated with resveratrol at a final concentration of 5 μM.

### Resveratrol May Rescue Cells Against H_2_O_2_ Induced Damage by Up-Regulating MEF2A

**Figure 2:**
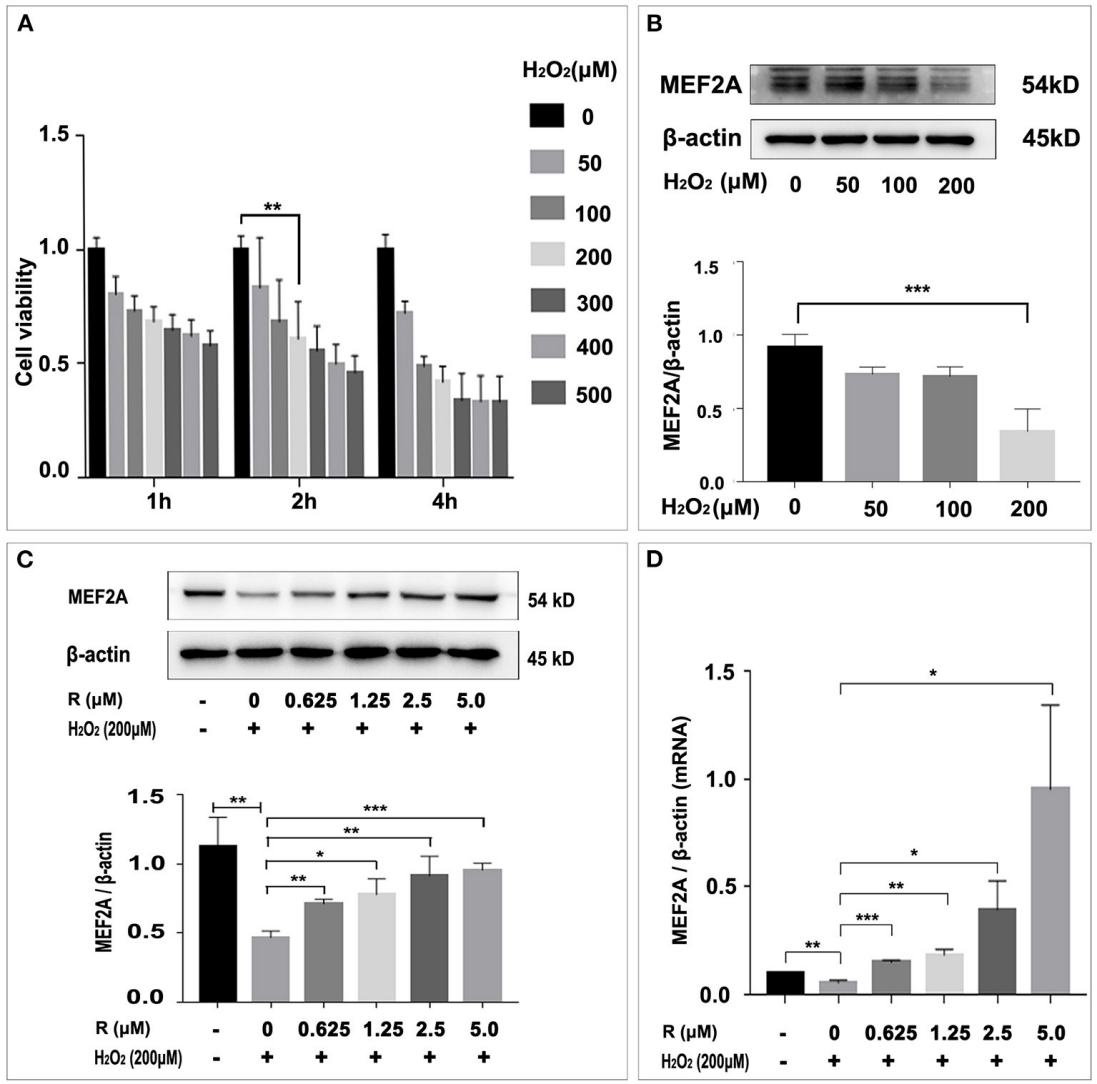
Influence of H_2_O_2_ on cell viability and resveratrol rescue MEF2A from the down-regulation induced by H_2_O_2_. **(A)** Effect of treatment of HUVEC with gradient concentration of H_2_O_2_ for various time on viability. **(B)** Immunoblots of MEF2A in HUVEC treated with gradient concentration of H_2_O_2_ for 2 h. **(C)** Expression of MEF2A proteins in HUVECs that were pretreated with gradient concentration of resveratrol for 24 h followed by treatment with H_2_O_2_ (200 μM for 2 h). β-actin was used as a loading control. Quantification of band intensity was performed via image-J and values were normalized to that of beta-actin. **(D)** The mRNA level of MEF2A in HUVECs that were pretreated with gradient concentration of resveratrol for 24 h followed by treatment with H_2_O_2_ (200 μM for 2 h). β-actin was used as the internal control to normalize the MEF2A mRNA level. All experiments were performed independently for three times. Mean ± SD is represented as bar plot. The statistical significance is analyzed by the unpaired Student's two-tailed *t*-test. ns, no significance; *P < 0.05; **P < 0.01; ***P < 0.001 between groups indicated.

In order to study the damage of H_2_O_2_ to HUVEC and its effect on MEF2A expression, we used different concentrations of H_2_O_2_ to treat HUVEC, to detect the changes of cell viability and MEF2A expression. The results showed that the cell damage caused by H_2_O_2_ was concentration-dependent. After treating HUVEC with 200 μM H_2_O_2_ for 2 h, the cell viability decreased about 40% (Figure 2A). A concentration-dependent reduction in the expression of MEF2A was observed as HUVECs were treated with H_2_O_2_. When HUVEC was treated with 200 μM H_2_O_2_ for 2 h, the protein expression level of MEF2A decreased about 60% (Figure 2B). In the follow-up experiments, 200 μM H_2_O_2_ was used to treat HUVEC for 2 h. The reduction of MEF2A protein or mRNA induced by H_2_O_2_ was abrogated by pretreating the cells with resveratrol, and the strength of this effect was dependent on the concentration of resveratrol (Figures 2C,D). It is interesting that the expression of SIRT1 altered in a similar pattern with MEF2A not only in the cells treated with a gradient concentration of resveratrol (Supplementary Figure 1A), but in the cells treated with a gradient concentration of H_2_O_2_ (Supplementary Figure 1B).

Our previous study has shown that overexpression of MEF2A can rescue H_2_O_2_ induced cell senescence (15). To investigate the damage effect of H_2_O_2_ or MEF2A specific siRNA (si-MEF2A) on HUVEC and whether resveratrol can rescue from the damage effect on HUVEC caused by H_2_O_2_ or si-MEF2A, we detected the changes in cell proliferation, mitochondrial membrane potential, apoptosis and the expression of apoptosis-related proteins after treatment of HUVEC with H_2_O_2_, si-MEF2A solely or plus resveratrol.

### Resveratrol May Prevent Cell Proliferative Ability Decrease Induced by H_2_O_2_ Through Up-Regulating MEF2A

**Figure 3:**
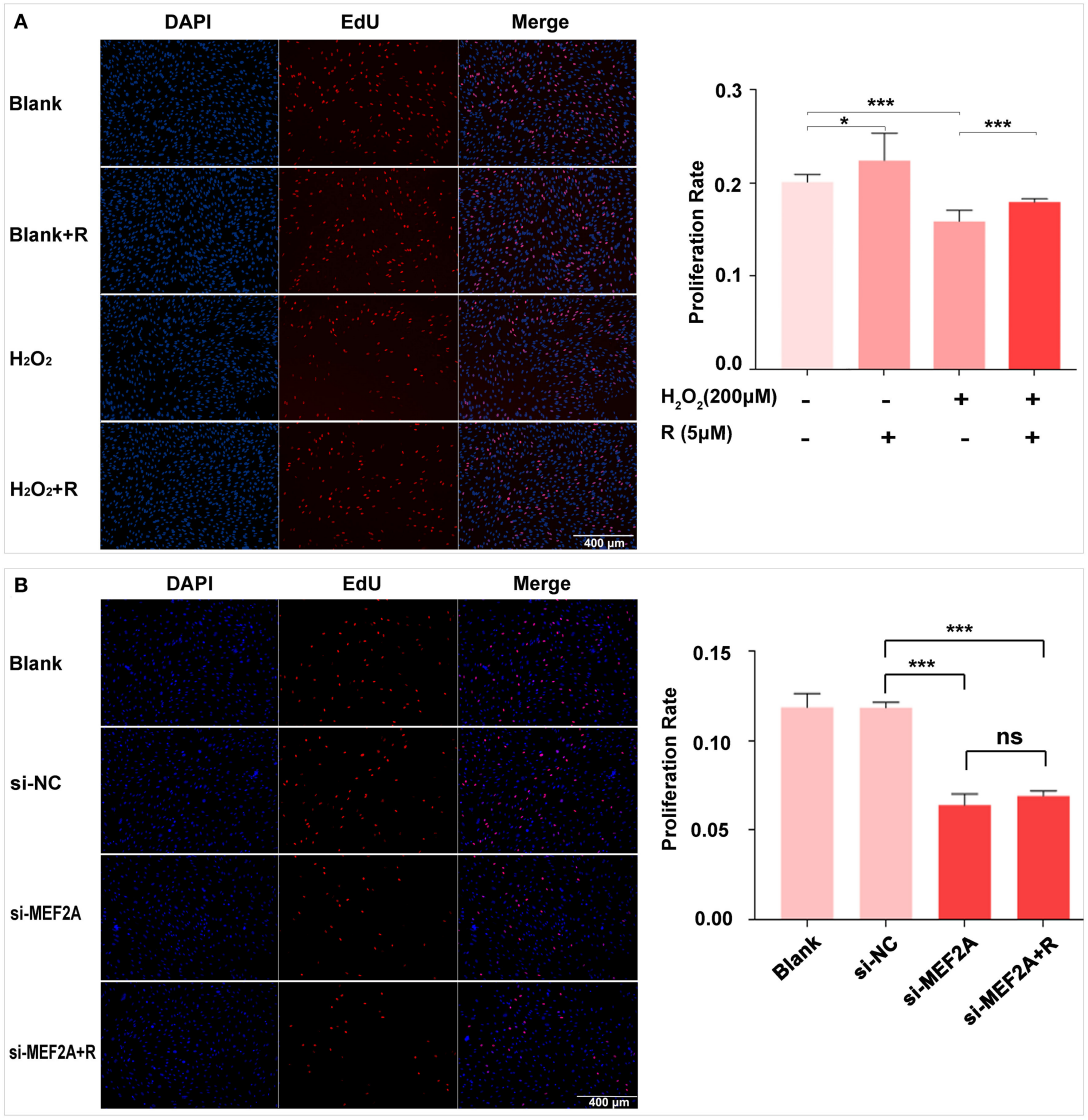
The impact of resveratrol on the decrease of cellular proliferation caused by treatment with H_2_O_2_ or by silencing MEF2A with siRNA. **(A)** Using EdU staining to test the impact of resveratrol on the cellular proliferation of HUVEC treated with or without H_2_O_2_. **(B)** Using EdU staining to detect the impact of resveratrol on the cellular proliferation of HUVEC caused by down-regulated MEF2A with siRNA. All experiments were performed independently for 3 times. R, resveratrol; Si-NC, the negative control of siRNA; Si-MEF2A, MEF2A specific siRNA. Mean ± SD is represented as bar plot. The statistical significance is analyzed by the unpaired Student's two-tailed *t*-test. ns, no significance; *P < 0.05; ***P < 0.001 between groups indicated.

EdU kit was used to detect the proliferative ability of cells in different treatment groups, and the results showed that the proliferative ability of HUVEC significantly reduced by treating with H_2_O_2_ or transfecting with si-MEF2A. Pretreatment of HUVEC with resveratrol before treating with H_2_O_2_ could prevent reduction of proliferative ability induced by H_2_O_2_ (Figure 3A). However, the decreased cell proliferative ability caused by si-MEF2A could not be reversed by treatment with resveratrol (Figure 3B). Silencing MEF2A with siRNA exerted more deleterious effect on the cellular proliferative ability than treatment of the cells with H_2_O_2_.

### Resveratrol May Rescue HUVEC Against H_2_O_2_ Induced Damage of Cellular Mitochondrial Membrane Upon Up-Regulating MEF2A

**Figure 4:**
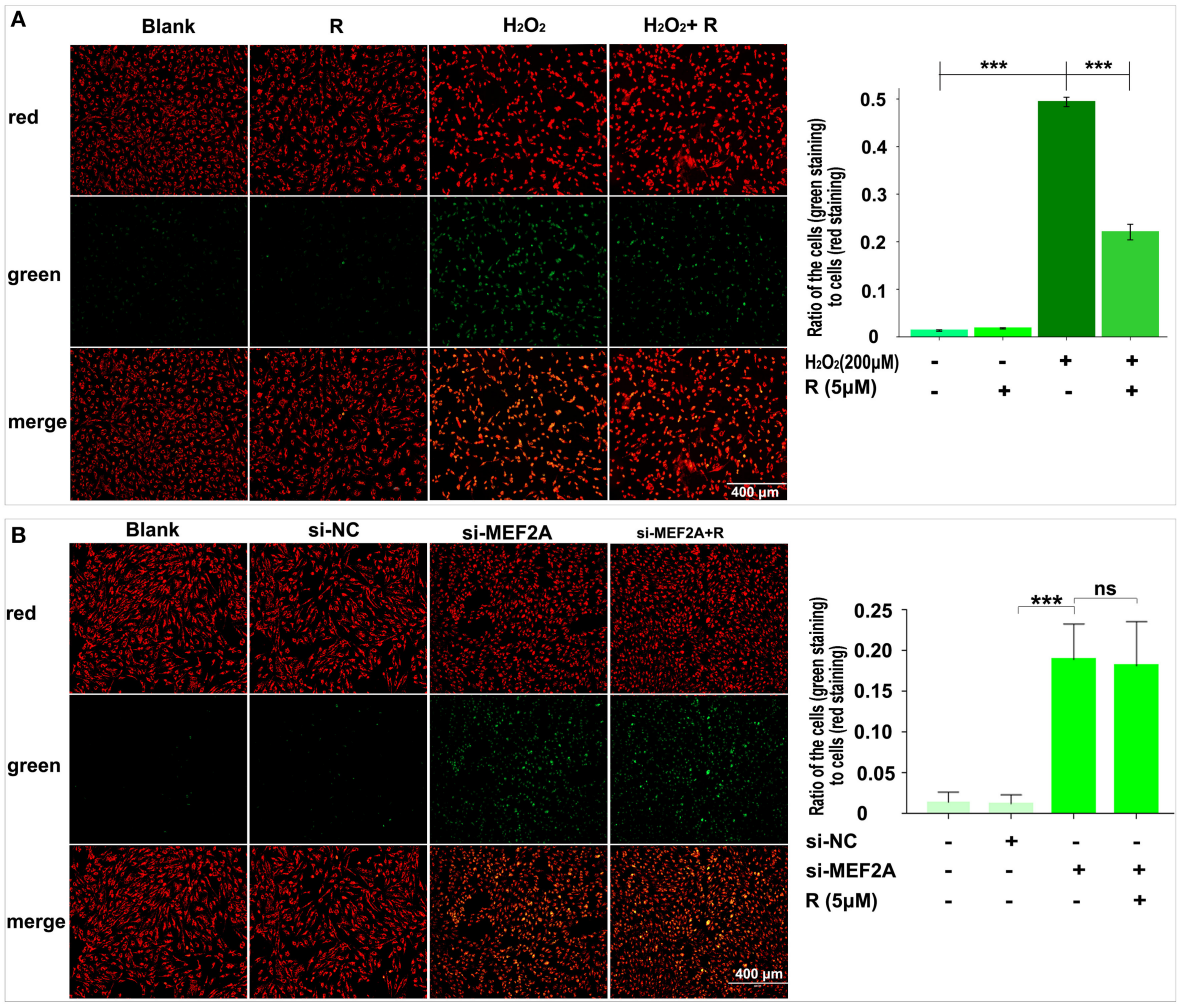
The impact of resveratrol on the decrease of mitochondrial membrane potential caused by treatment with H_2_O_2_ or by silencing MEF2A with siRNA. **(A)** Using JC-1 staining to test the impact of resveratrol on the cellular mitochondrial membrane potential of HUVEC treated with or without H_2_O_2_. **(B)** Using JC-1 staining to detect the impact of resveratrol on the cellular mitochondrial membrane potential of HUVEC caused by down-regulated MEF2A with siRNA. R, resveratrol; Si-NC, the negative control of siRNA; Si-MEF2A, MEF2A specific siRNA. All experiments were performed independently for 3 times. Mean ± SD is represented as bar plot. The statistical significance is analyzed by the unpaired Student's two-tailed *t*-test. ns, no significance; ***P < 0.001 between groups indicated.

The changes of the cell mitochondrial membrane potential were detected by JC-1 staining. The results showed that the proportion of green fluorescent cells increased significantly when HUVEC was treated with H_2_O_2_ (Figure 4A) or si-MEF2A (Figure 4B), indicating that treatment of the cells with H_2_O_2_ or si-MEF2A decreased the cell mitochondrial membrane potential and injured the cellular mitochondrial membrane. Pretreatment of HUVEC with resveratrol could decrease the ratio of the cells (green staining) to the cells (red staining), suggesting that resveratrol could rescue the cell from H_2_O_2_ induced mitochondrial membrane potential reduction (Figure 4A). However, the decrease of the cell mitochondrial membrane potential and the cell damage caused by si-MEF2A could not be reversed by treatment of cells with resveratrol (Figure 4B).

### Resveratrol May Prevent the Cell Apoptosis Induced by H_2_O_2_ Through Up-Regulating MEF2A

**Figure 5:**
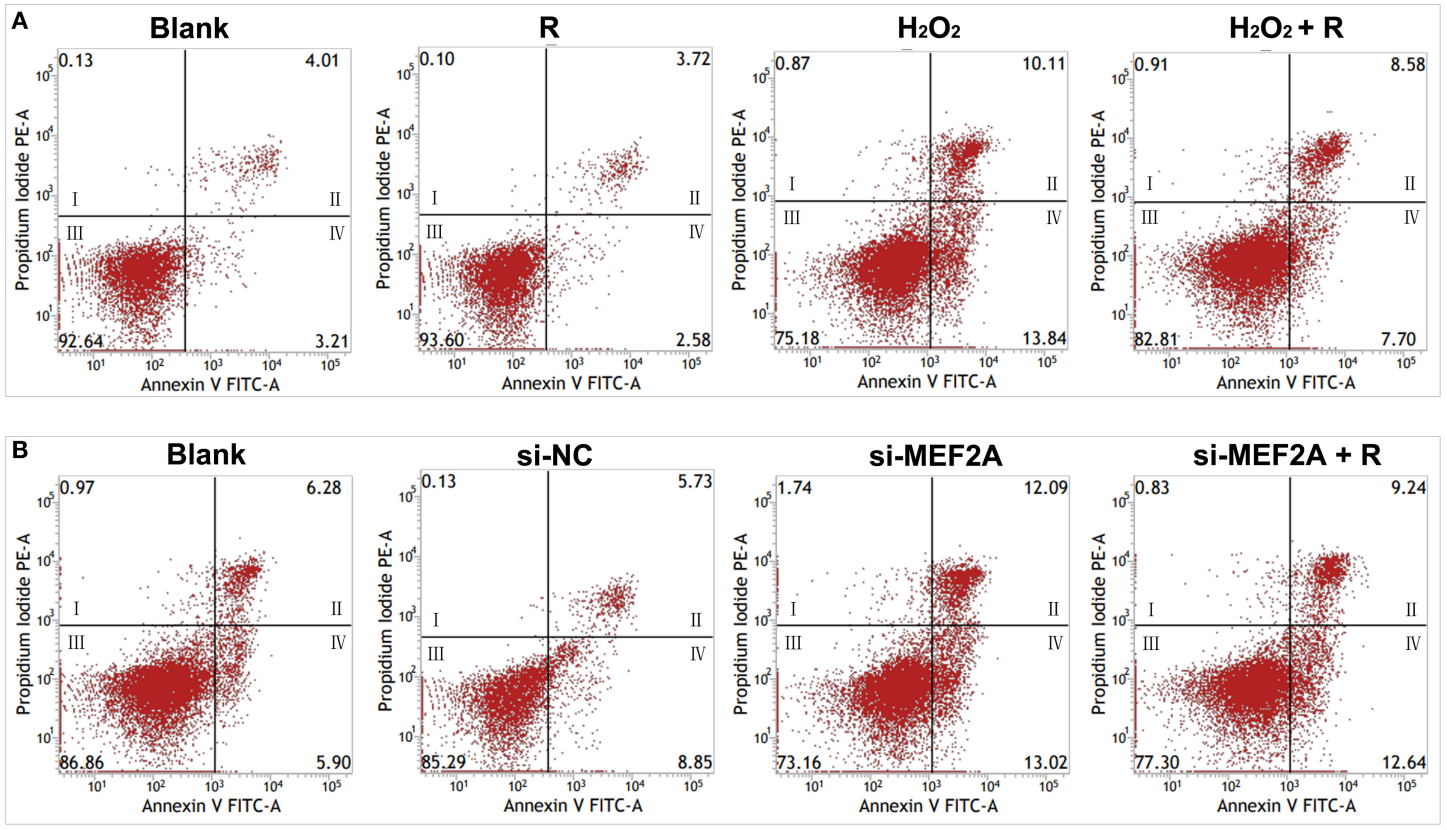
The impact of resveratrol on the apoptosis caused by the treatment with H_2_O_2_ or by silencing MEF2A with siRNA. **(A)** Using flow cytometry to test the impact of resveratrol on the apoptosis of HUVEC treated with or without H_2_O_2_. **(B)** Using flow cytometry to detect the impact of resveratrol on the apoptosis of HUVEC caused by down-regulated MEF2A with siRNA. R, resveratrol; Si-NC, the negative control of siRNA; Si-MEF2A, MEF2A specific siRNA. I: dead cells (Annexin V-FITC^−^/PI^+^); II: late apoptotic/necrotic cells (Annexin V-FITC^+^/PI^+^); III: non-apoptotic cells (Annexin V-FITC^−^/PI^−^); IV: early apoptotic cells (Annexin V-FITC^+^/PI^−^).

Flow cytometry was used to detect the apoptosis rate and the results showed that the number of apoptotic cells significantly increased in the group of treatment with H_2_O_2_ (early apoptotic cells: 13.84 vs. 3.21% and late apoptotic/necrotic cells: 10.11 vs. 4.01%) or si-MEF2A (early apoptotic cells: 13.02 vs. 8.85% and late apoptotic/necrotic cells: 12.09 vs. 5.73%) respectively compared with that in the blank group and the si-NC group. Pretreatment of the cells with resveratrol before treating the cells with H_2_O_2_ partially rescued the cells from apoptosis (Figure 5A) (early apoptotic cells: 7.70 vs. 13.84% and late apoptotic/necrotic cells: 8.58 vs. 10.11%), but the apoptosis caused by si-MEF2A could not be improved by treatment of the cells with resveratrol (Figure 5B) (early apoptotic cells: 12.64 vs. 13.02% and late apoptotic/necrotic cells: 12.09 vs. 9.24%).

### Resveratrol May Prevent Apoptosis-Related Proteins to Be Altered by Treatment of the Cells With H_2_O_2_ on Up-Regulating MEF2A

**Figure 6:**
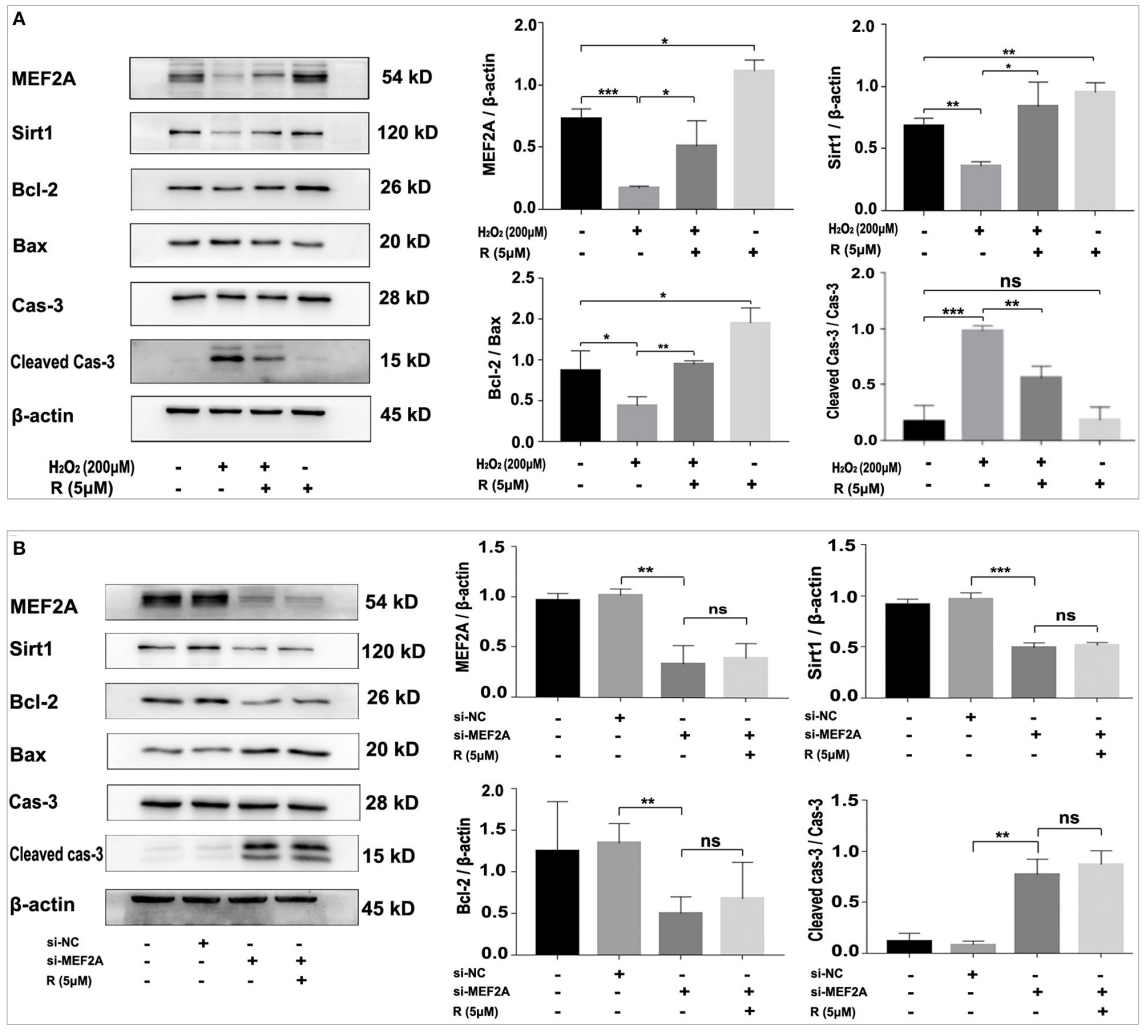
Resveratrol eliminates the alteration of apoptosis-related proteins caused by treatment with H_2_O_2_ but not with down-regulation of MEF2A with siRNA. **(A)** Immunoblots of MEF2A and apoptosis-related proteins for the groups that were treated with H_2_O_2_ and resveratrol, solely or together. **(B)** Using immunoblots to detect alteration of the apoptosis-related proteins caused by silencing MEF2A with siRNA and treatment with resveratrol. Quantification of the band intensity was performed via image-J and values were normalized to beta-actin. R, resveratrol; Cas-3, caspase-3; Si-NC, the negative control of siRNA; Si-MEF2A, MEF2A specific siRNA. All experiments were performed independently for three times. Mean ± SD is represented as bar plot. The statistical significance is analyzed by the unpaired Student's two-tailed *t*-test. ns, no significance; *P < 0.05; **P < 0.01; ***P < 0.001 between groups indicated.

Treatment of HUVEC with resveratrol promoted the expression of MEF2A, Sirt1 and Bcl-2, and down-regulated pro-apoptotic proteins such as Bax and cleaved caspase-3, while H_2_O_2_-treated HUVEC significantly down-regulated the protein levels of MEF2A, Sirt1 and Bcl-2, up-regulated pro-apoptotic proteins such as Bax and cleaved caspase-3 (Figure 6A). Pretreatment of HUVEC with resveratrol before treating the cells with H_2_O_2_ could rescue the expression of the apoptosis-related proteins altered by H_2_O_2_. These results suggest that resveratrol prevent H_2_O_2_-induced apoptosis may be via up-regulating the expression of MEF2A. The expression of SIRT1 not only changed with MEF2A at the protein levels (Figure 6), but with MEF2A at the mRNA levels (Supplementary Figure 1), which suggested that MEF2A may regulate SIRT1 transcriptionally.

Transfection of HUVEC with si-MEF2A significantly down-regulated the protein levels of Sirt1 and Bcl-2, and up-regulated pro-apoptotic proteins such as Bax and cleaved caspase-3, which could not be abrogated by treatment of the cells with resveratrol (Figure 6B). These results demonstrated at the molecular level that MEF2A has anti-apoptotic function and the promotion of SIRT1 expression by resveratrol is dependent on the expression of MEF2A, further suggesting that the anti-apoptotic function of resveratrol is realized via promoting the expression of MEF2A.

### Damaged Vascular Endothelial Integrity Was Observed in Vascular Endothelium of the MEF2A Silenced Mice

**Figure 7:**
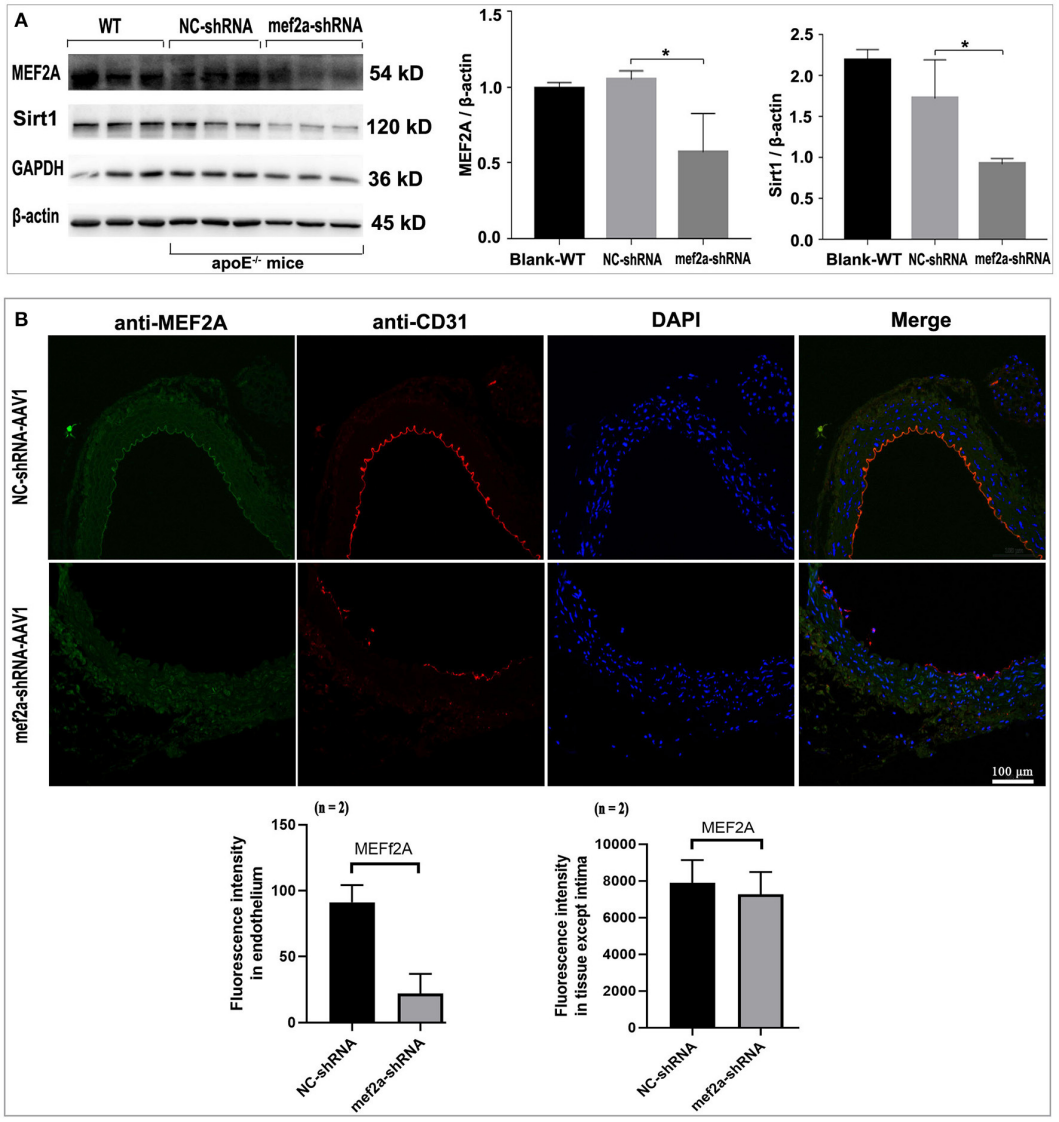
Expression of MEF2A, SIRT1 and CD31 in the vessels of the mice. **(A)** Immunoblots of MEF2A and SIRT1. Mean ± SD is represented as bar plot. The statistical significance is analyzed by the unpaired Student's two-tailed *t*-test. ns, no significance; *P < 0.05 between groups indicated. **(B)** Immunofluorescence of MEF2A and CD31 in the vascular endothelium. The cells expressed sufficient MEF2A were stained green, and the cells expressed sufficient CD31 were stained red. WT: wild type mice; The “n” indicates the sample size.

The immunoblotting results showed that the expression of MEF2A in the vascular tissue from mef2a-shRNA-AAV1 group was significantly lower than that from NC-shRNA-AAV1 group (*n* = 3) (Figure 7A), suggesting that si-MEF2A AAV1 effectively knocked down the expression of MEF2A. There was no significant difference in the protein level of MEF2A in myocardial tissues from different groups (*n* = 3) (Supplementary Figure 2), indicating that MEF2A-shRNA may only specifically silence MEF2A in vascular tissues, which is consistent with the specificity speculated according to that the promoter of endothelial cell specific expression gene (ICAM2) was used construct the adeno-associated virus vector to control the expression of MEF2A specific shRNA. The expression of SIRT1 in vascular tissue was also significantly down-regulated in mef2a-shRNA-AAV1 group (Figure 7A), indicated that SIRT1 is regulated by MEF2A. The expression of MEF2A in vascular endothelium was detected by immunofluorescence and the results showed that the fluorescence intensity of si-MEF2A group was significantly weaker than that of the si-NC group (*n* = 2) (Figure 7B). CD31, a specific marker of vascular endothelium, was detected by immunofluorescence. The results showed that there were continuous fluorescent bands along the inner edge of blood vessels in si-NC group, while there was only intermittent fluorescent staining area along the inner edge of blood vessels in mef2a-shRNA-AAV1 group, suggesting that the integrity of vascular endothelium might be destroyed after interfering with MEF2A (Figure 7B).

Knocked-down the MEF2A in the vascular endothelium decreased the expression of SIRT1 and increased the cleaved caspase-3 level in the vascular tissue, and the alteration of SIRT1 and cleaved caspase-3 could not be reversed when the apoE^−/−^ mice have been subjected to gastric administration of resveratrol (Supplementary Figure 3).

### Atherosclerotic Lesion Was Alleviated by Gastric Administration of Resveratrol in ApoE^–/–^ Mice

Oil red O staining for the aorta (*n* = 1) (Supplementary Figure 4A) and the aortic valve (*n* = 2–4) (Supplementary Figure 4B) showed that the lipid deposition in the aorta and aortic valve of apoE^−/−^ mice was serious than that in wild type mice, the lipid deposition in the aorta and aortic valve of the mice of mef2a-shRNA-AAV1 groups were more severe than those of NC-shRNA-AAV1 groups. Gastric administration of resveratrol showed a trend to alleviate lipid deposition in both NC-shRNA-AAV1 and mef2a-shRNA-AAV1 (Supplementary Figures 4A-1,B-1).

### Effects of Feeding With Resveratrol and Interfering With MEF2A on Lipid Metabolism in ApoE^–/–^ Mice

**Figure 8:**
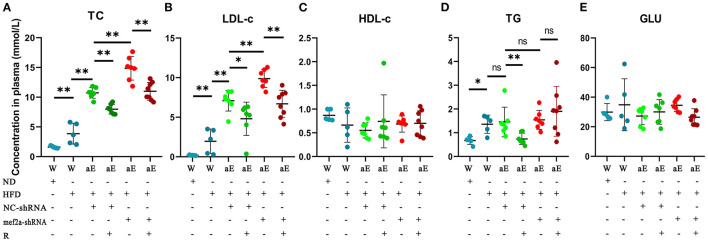
The influence of gastric administration of resveratrol or silencing MEF2A in vascular endothelium of apoE^−/−^ mice on plasma levels of cholesterol, lipid and glucose. **(A)** Total cholesterol (TC). **(B)** Low-density lipoprotein cholesterol (LDL-c). **(C)** High-density lipoprotein cholesterol (HDL-c). **(D)** Triglyceride (TG). **(E)** Glucose (GLU). W: wild type mice; aE: apoE^−/−^ mice; ND, normal diet; HFD, high-fat diet; NC-shRNA, negative control (infection with NC-shRNA-AAV1); mef2a-shRNA, infection with mef2a-shRNA-AAV1; R, resveratrol; “-” indicates no treatment performed with the factor at the line; “+” indicates treatment performed with the factor at the line. The individual values are presented in dot plots and the values distribution is represented as mean ± standard deviation (SD). The statistical significance is analyzed by the Mann-Whitney U test. ns, no significance; *P < 0.05; **P < 0.01 between groups indicated.

Blood samples were collected and five biochemical indicators including total cholesterol (TC), low density lipoprotein cholesterol (LDL-C), high density lipoprotein cholesterol (HDL-C), triglyceride (TG) and glucose (GLU) were detected and the quantitative and statistical significance test results were respectively showed in Supplementary Tables 1, 2. The levels of plasma lipids of wild-type mice fed with high-fat diet were significantly higher than those of wild-type mice fed with normal diet (*n* = 8) (Figure 8), indicating an actual effect of the high-fat diet on the levels of the blood lipids. The levels of plasma lipids of all the apoE^−/−^ mice were significantly higher than those of wild-type mice (Figure 8), suggesting that apoE^−/−^ mice have abnormal lipid metabolism. Knockdown of MEF2A in the vascular endothelium led to a significant increase of plasma TC (Figure 8A) and LDL-c (Figure 8B). Significantly decreased LDL-c and TC was observed in both NC-shRNA and mef2a-shRNA mice that were subjected to gastric administration of resveratrol. TG was remarkably reduced by gastric administration of resveratrol in si-NC mice but not in si-MEF2A mice (Figure 8D). Gastric administration of resveratrol and knockdown of MEF2A did not affect the plasma levels of HDL-c (Figure 8C) and GLU (Figure 8E). These results suggest that MEF2A plays an important role in lipid metabolism. The function of resveratrol to reduce LDL-C is independent of MEF2A, while the function of resveratrol to reduce TG may depend on MEF2A.

## Discussion

In this study, we revealed for the first time that the protective effect of resveratrol on vascular endothelial cell depended on promoting the expression of MEF2A to activate the expression of SIRT1, thereby enhanced the ability of the cells to resist oxidative stress and anti-apoptosis. In addition, down-regulated expression of MEF2A in the vascular endothelium of apoE^−/−^ mice affected the lipid metabolism, leading to a significant increase in plasma LDL-c level. Regardless of whether knocked down the expression of MEF2A in mice, resveratrol significantly reduced LDL-c and TG. However, the function of resveratrol to reduce TG in apoE^−/−^ mice depended on the sufficient expression of MEF2A.

We also observed that knockdown of MEF2A in HUVEC had similar effects on cells as treatment of the cells with H_2_O_2_, such as reducing cell viability and mitochondrial membrane potential, promoting apoptosis and so on. These indicate that the harmful effect of H_2_O_2_ on the cells may be mainly attributed to a significant down-regulation of MEF2A induced by H_2_O_2_. However, pretreatment of HUVEC with resveratrol could prevent the down-regulation of MEF2A induced by H_2_O_2_, thus prevented the effects of H_2_O_2_ on cell viability, mitochondrial membrane potential and apoptosis, which further supports the important role of MEF2A in anti-oxidative stress. In HUVEC of silencing MEF2A with siRNA, treatment of the cells with resveratrol could not restore the expression of MEF2A, also not abrogate or alleviate the detrimental influence of silencing MEF2A on the cells, suggesting that the protective function of resveratrol on vascular endothelial cells depends on its role in promoting the expression of MEF2A. Liu et al. (15) also reported that treatment of HUVEC with H_2_O_2_ significantly down-regulated the expression of MEF2A and promoted cell senescence, but overexpression of MEF2A in HUVEC ensured that there was still a high expression of MEF2A in H_2_O_2_-treated cells, thus prevented cell senescence induced by H_2_O_2_. The mechanism is that MEF2A upregulates the expression of SIRT1 through PI3K/p-AKT1 pathway. The main characteristics of cell senescence are mitochondrial dysfunction, genomic instability, aging-related secretory phenotype (SASP), epigenome changes and stem cell exhaust (28). Silencing MEF2A in HUVEC led to a significant decrease in mitochondrial membrane potential, showing the characteristics of cell senescence or apoptosis. Treatment of HUVEC with resveratrol was similar to overexpression of MEF2A in the cells, exhibited a strong anti-oxidative stress feature. All the above suggest that MEF2A plays an important regulatory role in both anti-apoptosis and anti-aging, and the anti-apoptosis and anti-aging effects of resveratrol mainly depends on its role in up-regulation of MEF2A expression.

Up-regulation of SIRT1 is considered to be an important molecular mechanism for resveratrol to exert its cytoprotective function. Resveratrol up-regulates the expression of nitric oxide synthase (eNOS) and enzyme activity in vascular endothelial cells by up-regulating the expression of SIRT1, and increases the NO production to mediate vasodilation of the blood vessel (1, 29). Resveratrol can enhance the ability of autophagy by activating Sirt1/Foxo1 pathway, prevent oxidative stress and apoptosis induced by H_2_O_2_ (30, 31), and prevent apoptosis and oxidative stress induced by ischemia/reperfusion (32). Resveratrol also improved endothelial dysfunction in obese and diabetic mice through SIRT1/PPAR pathway, and improved glucose absorption in adipocytes with insulin resistance (33). Resveratrol improves rheumatoid arthritis by activating SIRT1-Nrf2 signaling pathway and autophagy (34, 35), and plays a beneficial role in mitochondrial function by activating AMPK (36). In this study, we once again observed that resveratrol has a significant upregulation effect on SIRT1 in HUVEC, but the upregulation of SIRT1 by resveratrol depended on its role in upregulation of MEF2A. When the expression of MEF2A was silenced by siRNA and could not be restored by resveratrol *in vivo* or *in vitro*, resveratrol could not up-regulate SIRT1 and also lost its protective role, which strongly indicates that promoting the expression of MEF2A is a more critical and upstream event for resveratrol to exert its cytoprotective function. In previous studies, we have demonstrated that MEF2A regulates the expression of SIRT1 (15), which was consolidated by the experimental evidence from *in vitro* and *in vivo* in this study.

Resveratrol was failure to restore or promote the expression of MEF2A silenced by siRNA in vascular endothelial cells, which might be the major reason for that resveratrol lost its protection against atherosclerosis in MEF2A-silenced apoE^−/−^ mice. The protective effect of MEF2A on blood vessels has also been sporadically reported in previous studies. Zhou et al. (25) reported that inhibition of MEF2A with siRNA can accelerate atherosclerosis in apoE^−/−^ mice, and its molecular mechanism is involved in the increase of pro-inflammatory cytokines such as MCP-1, MMP-8, IL-6 and TNF-α. Lu et al. reported that the deletion of MEF2A in mice is more likely to lead to an increase in the risk of bleeding, hypercoagulability and inflammation (23). Xiong et al. (27) showed that interfering MEF2A in HCAEC significantly affects the gene expression profile of inflammation-related pathways. Except anti-aging, anti-apoptosis and anti-oxidative stress, SIRT1 that is confirmedly regulated by MEF2A in our studies, also plays an important role in the anti-inflammatory pathway (37). These results suggest that MEF2A may also exert the protective function on vascular endothelial cells by regulating the inflammatory pathway. The integrity of the vascular endothelium was damaged in MEF2A knocked down mice, which may be attributed to the comprehensive effect of silencing MEF2A in vascular endothelial cells, such as acceleration of aging, promotion of apoptosis, reduction of anti-oxidative stress and anti-inflammation.

It's very interesting that knockdown of MEF2A in vascular endothelium significantly increased the level of plasma LDL-c, which has not been reported in previous studies. Although many functions of resveratrol seemingly disappeared while the expression of MEF2A was knocked down by siRNA, the role of resveratrol in reducing LDL-c was unaffected in MEF2A-knocked down mice. Previous studies have shown that resveratrol can significantly reduce LDL-c and TG (38). Yet we found that knockdown of MEF2A in the vascular endothelium could not increase TG, but apparently eliminated the role of resveratrol in decreasing TG. These results suggest that MEF2A may be involved in the production and metabolism of LDL-c and TG with different manner. Involvement of MEF2A in the lipid metabolism pathway has been reported in several studies. Zhao et al. (39) found that specifically reducing the expression of MEF2 in drosophila muscle leads to the accumulation of large amounts of cholesterol-rich lipid fluid in muscle when fed high-calorie or carbohydrate-rich foods. GLUT4 promotes lipid metabolism and the expression of GLUT4 is regulated by MEF2A (40, 41), suggesting that the regulation of lipid metabolism by MEF2A may be related to GLUT4. SIRT1 increases liver LDL receptor (LDLR) by inhibiting the secretion of proprotein convertase subtilisin/kexin 9 (PCSK9), thus reducing plasma LDL-C level (42). Considering the above previous study, increase in plasma LDL-c by silencing MEF2A may be through regulating SIRT1/PCSK9/LDLR pathway. However, in MEF2A knocked down mice, resveratrol could not up-regulate Sirt1, still reduce plasma LDL-c but not plasma TG, suggesting that resveratrol can also activate other lipid reducing pathways independent of MEF2A/Sirt1/Pcsk9/LDLR, and its effect on reducing TG may depend on activating MEF2A/SIRT1 pathway. In the MEF2A interference group, resveratrol also showed the trend of preventing lipid deposition, which also indicate that there is a LDL-C reducing effect of resveratrol independent of MEF2A *in vivo*. Although there is progression in studies that can help to interpret the involvement of MEF2A in lipid metabolism, but the associated molecular mechanism is obscure and should be investigated. How does MEF2A participate in the promotion of TG metabolism by resveratrol is also worthy further studied in future.

Although the evidences acquired consistently support that the protective effect of resveratrol on vascular endothelial cells depends on activating the expression of MEF2A, but there are some limitations in *in-vivo* experiments of this study. (1) The number of samples for oil red O staining to detect lipid deposition in the whole aortic vessel was only one sample. However, the change trend of lipid deposition observed in the oil red O staining experiment was very consistent with that of LDL-C in plasma (*n* = 8), which indirectly proved that the experimental results of oil red O were relatively reliable. (2) Only two samples were used to detect the effectiveness and specificity of MEF2A knockdown by immunofluorescence. (3) The mechanism of resveratrol up-regulating MEF2A and the molecular mechanism of MEF2A protecting vascular endothelium were not deeply studied. (4) We observed the destruction of endothelial integrity by silencing MEF2A *in vivo*, but didn't further study on its mechanism. Therefore, the association of resveratrol and MEF2A with vascular protection *in vivo* needs to be further studied in many aspects.

In summary, MEF2A is involved in the normal physiological process of cells, especially in maintaining the function of vascular endothelial cells. Therefore, enhancing or maintaining the expression of MEF2A in vascular endothelial cells may be a novel strategy to develop vascular protective methods or explore vascular protective drugs, and also provides a novel perspective for further research on the molecular mechanism of the occurrence and development of cardiovascular diseases. Resveratrol, that is a widely demonstrated cardiovascular protective drug, has been considered to function through activate SIRT1 pathway in previous studies. Here, we revealed a novel mechanism involved in the protective role of resveratrol, is that promotion of the expression of MEF2A is required for the protective role of resveratrol in vascular endothelial cells.

## Conclusion

Protection of resveratrol on VEC depends on up-regulating the expression of MEF2A, and the main mechanism may be that up-regulation of MEF2A can promote the expression of anti-apoptosis and anti-aging related genes such as SIRT1, and enhance the ability of cells to resist oxidative stress, thus inhibit premature apoptosis or senescence of vascular endothelial cells. MEF2A may also play an important role in maintaining lipid metabolism homeostasis of vascular endothelium.

## Data Availability Statement

The raw data supporting the conclusions of this article will be made available by the authors, without undue reservation.

## Ethics Statement

The animal study was reviewed and approved by the Experimental Animal Ethics Committee of the Second Affiliated Hospital of Guangzhou Medical University.

## Author Contributions

All authors listed have made a substantial, direct, and intellectual contribution to the work and approved it for publication.

## Funding

This study was supported by Guangzhou Science and Technology Project (202102010054 to BL) and the General Programs of the National Natural Science Foundation of China (81873474 to SML).

## Conflict of Interest

The authors declare that the research was conducted in the absence of any commercial or financial relationships that could be construed as a potential conflict of interest.

## Publisher's Note

All claims expressed in this article are solely those of the authors and do not necessarily represent those of their affiliated organizations, or those of the publisher, the editors and the reviewers. Any product that may be evaluated in this article, or claim that may be made by its manufacturer, is not guaranteed or endorsed by the publisher.
