# Supplementary material for: MEF2A Is the Trigger of Resveratrol Exerting Protection on Vascular Endothelial Cell
Source: Front Cardiovasc Med. 2022 Jan 3;8:775392. doi: 10.3389/fcvm.2021.775392 (PMC8762055; doi:10.3389/fcvm.2021.775392)

Supplemental Materials

Table S1. Comparison of blood lipid and blood glucose among groups.

Group No.		TC (mmol/L)	HDL_C (mmol/L)	LDL_C (mmol/L)	TG (mmol/L)	GLU (mmol/L)
**0**	**W+ND**	1.606±0.201	0.87±0.119	0.19±0.039	0.672±0.162	29.92±5.81
**1**	**W+HFD**	3.862±1.746	0.664±0.362	1.962±1.547	1.36±0.384	34.866±17.583
**2**	**aE+NC-shRNA**	10.717±0.887	0.553±0.16	7.081±1.297	1.456±0.623	27.246±5.813
**3**	**aE+NC-shRNA+R**	7.97±0.81	0.743±0.558	4.801±2.1	0.741±0.234	30.02±8.311
**4**	**aE+mef2a-shRNA**	14.856±1.988	0.687±0.172	9.887±1.136	1.543±0.399	34.509±4.166
**5**	**aE+mef2a-shRNA+R**	10.998±1.499	0.7±0.265	6.698±1.707	1.904±1.053	26.446±5.743

Note: mean ± standard deviation; W: wild type mice; aE: apoE$^{-/-}$ mice; ND: normal diet; HFD: high-fat diet; NC-shRNA: negative control (infection with NC-shRNA-AAV1); mef2a-shRNA: infection with mef2a-shRNA-AAV1; R: resveratrol.

Table S2. Statistical significance between any tow groups was tested with Student's t test.

Group pairs	*P* value				
	TC	HDL_C	LDL_C	TG	GLU
(0, 1)	0.0208	0.2617	0.0336	0.0062	0.5669
(0, 2)	< 0.0001	0.0039	< 0.0001	0.0217	0.4502
(0, 3)	< 0.0001	0.6311	0.0007	0.5820	0.9821
(0, 4)	< 0.0001	0.0689	< 0.0001	0.0010	0.1401
(0, 5)	< 0.0001	0.2087	< 0.0001	0.0267	0.3134
(1, 2)	< 0.0001	0.4832	0.0001	0.7683	0.3035
(1, 3)	0.0003	0.7886	0.0286	0.0059	0.5340
(1, 4)	< 0.0001	0.8845	< 0.0001	0.4455	0.9590
(1, 5)	< 0.0001	0.8393	0.0004	0.2970	0.2273
(2, 3)	0.0001	0.4033	0.0309	0.0149	0.4831
(2, 4)	0.0003	0.1571	0.0010	0.7605	0.0198
(2, 5)	0.6729	0.2245	0.6362	0.3436	0.7933
(3, 4)	< 0.0001	0.8049	0.0001	0.0006	0.2256
(3, 5)	0.0004	0.8488	0.0757	0.0137	0.3449
(4, 5)	0.0009	0.9145	0.0011	0.4098	0.0090

Note: The number in the column 'group pairs' is corresponding to that of Group No. In Table S1.

Supplemental Figures

Figure S1. Alteration of the mRNA level of SIRT1 as treatment of HUVEC with resveratrol or H_2O_2. **(A)** The mRNA level of SIRT1 in HUVEC treated with gradient concentration of resveratrol for 24 hours. **(B)** The mRNA level of SIRT1 in HUVECs that were pretreated with gradient concentration of resveratrol for 24 hours followed by treatment with H_2O_2 (200 μ M for 2 hours). All experiments were performed independently for 3 times. β -actin was used as the internal control to normalize the SIRT1 mRNA level. Mean ± SD is represented as bar plot. The statistical significance is analyzed by the unpaired Student's two-tailed t-test. R, resveratrol; *, $P < 0.05$; **, $P < 0.01$; ***, $\boldsymbol{P} < 0.001$ between groups indicated.

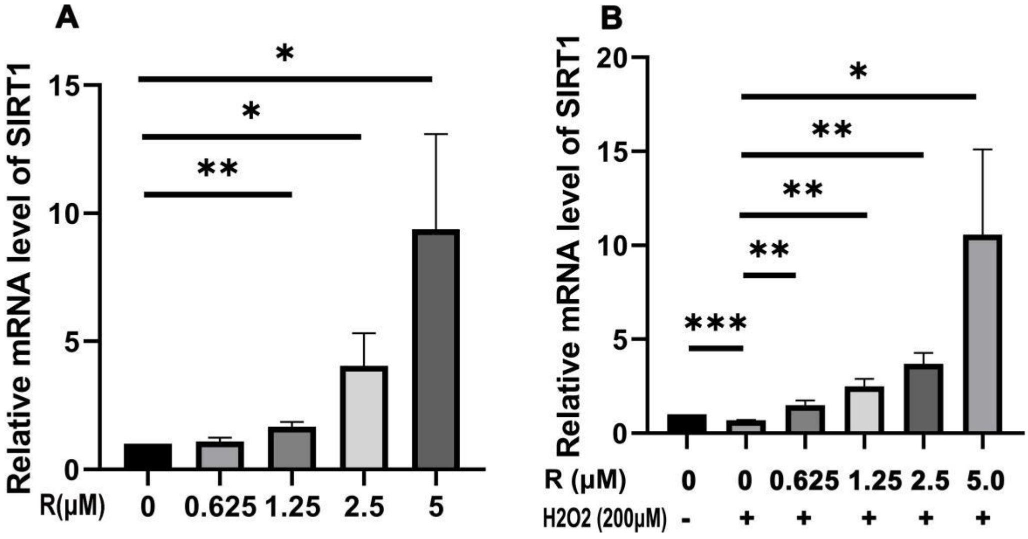

Figure S2. Proteins in myocardial tissues from different groups of apoE$^{-/-}$ mice.

N = 3； NC-shRNA: the groups of mice infected with negative control shRNA AAV1; mef2a-shRNA: the groups of mice infected with MEF2A specific interfering shRNA AAV1; R: resveratrol. '+ R' indicates that mice in this group were subjected to gastric administration of resveratrol. 'ns' indicates no significance.

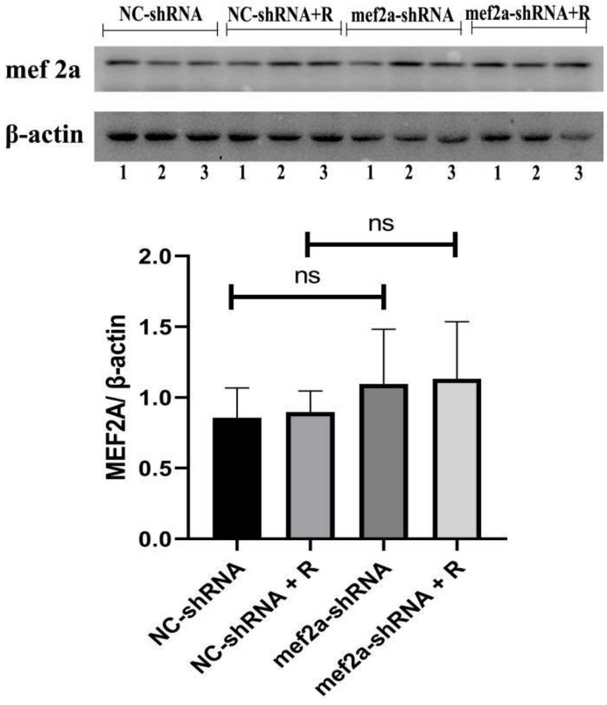

Figure S3. Alteration of proteins in vascular tissues from different groups of apoE$^{-/-}$ mice.

N = 3； NC-shRNA: the groups of mice infected with negative control shRNA AAV1; mef2a-shRNA: the groups of mice infected with MEF2A specific interfering shRNA AAV1; R: resveratrol; cleaved cas-3: cleaved caspase-3.

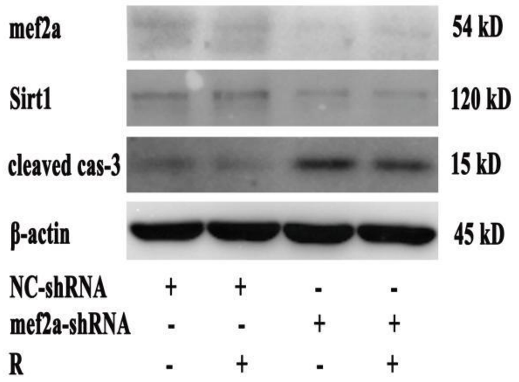

Figure S4. The influence of gastric administration of resveratrol and silencing MEF2A in vascular endothelium of $apoE^{-/-}$ mice on the lipid deposition. (A) Whole thoracic aorta was stained with oil red O (n = 1). (B) Sections of aortic valve were stained with oil red O (n = 2-4). (A-1) The percentage of the plaque area in the whole aorta semi-quantified with image J.(B-1) The percentage of the plaque area in aortic valve semi-quantified with image J, the numbers on the bars indicate the sample size analyzed. The red staining area are the lipid deposition area. R, resveratrol; WT, wild type; $apoE^{-/-}$, $apoE^{-/-}$ mice; ND, normal diet; HFD, high-fat diet. *, $P < 0.05$.

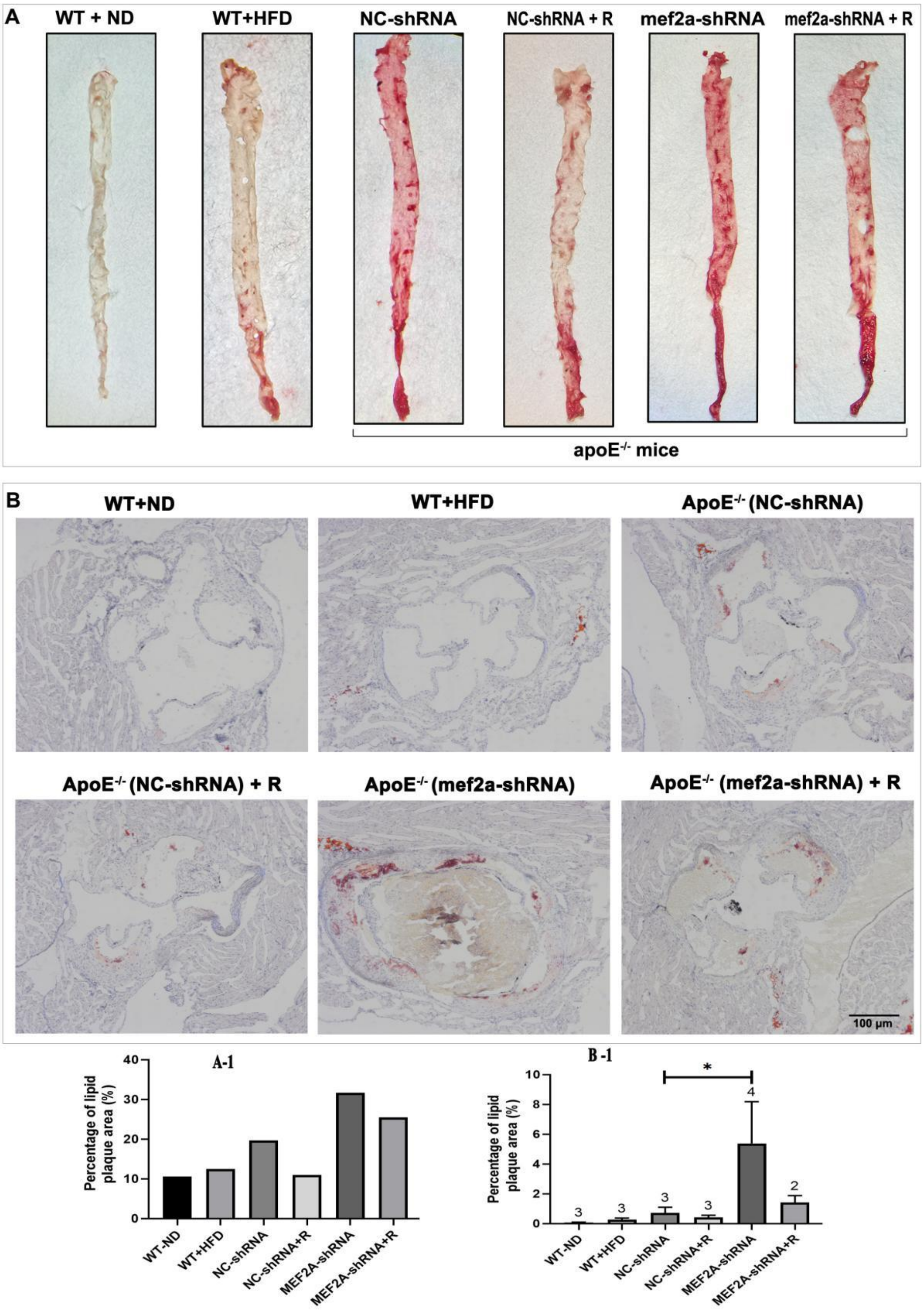

Supplement: Supplementary file 1 [file Data_Sheet_1.PDF]
