# Supplementary figures and images for: MEF2A Is the Trigger of Resveratrol Exerting Protection on Vascular Endothelial Cell
Source: Front Cardiovasc Med. 2022 Jan 3;8:775392. doi: 10.3389/fcvm.2021.775392 (PMC8762055; doi:10.3389/fcvm.2021.775392)

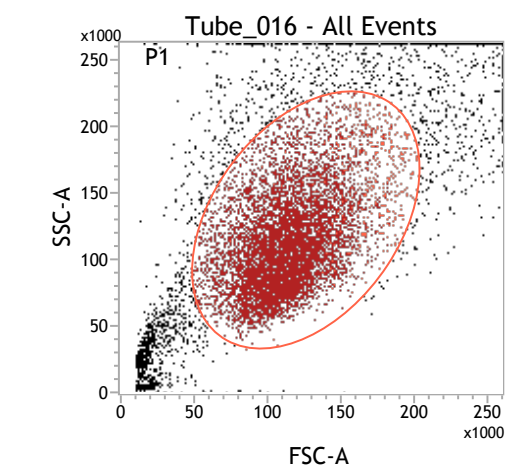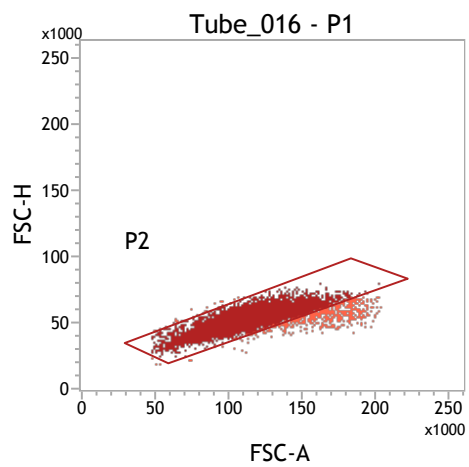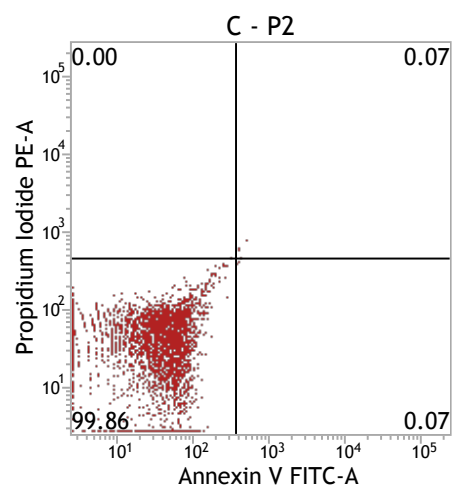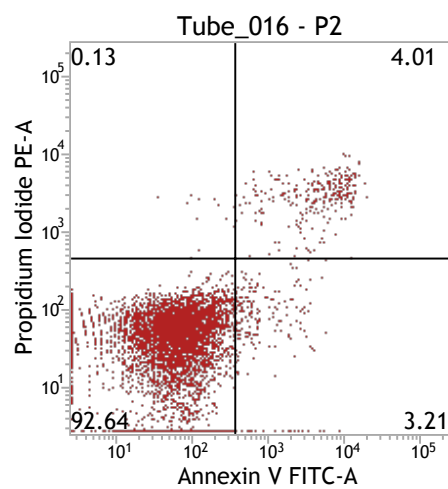

Blank

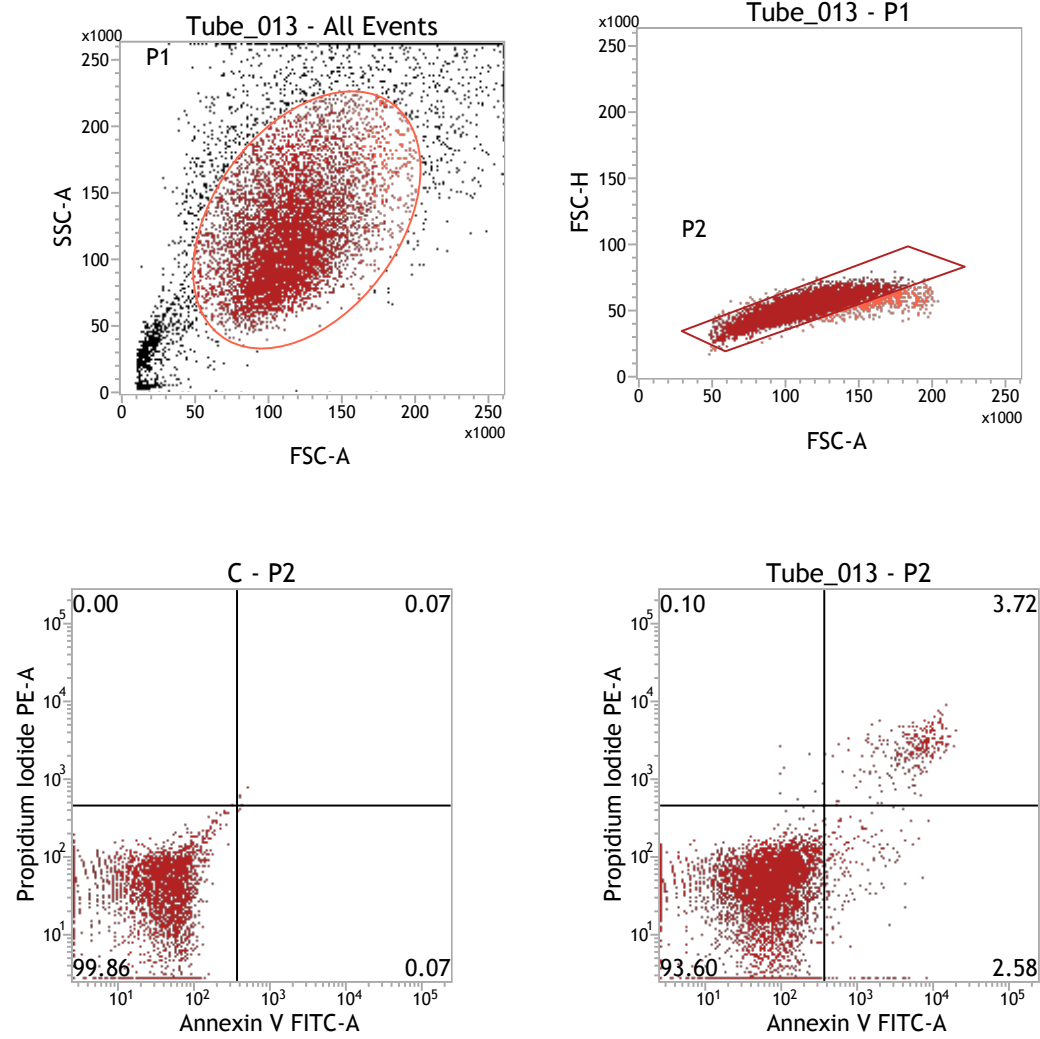

Resveratrol

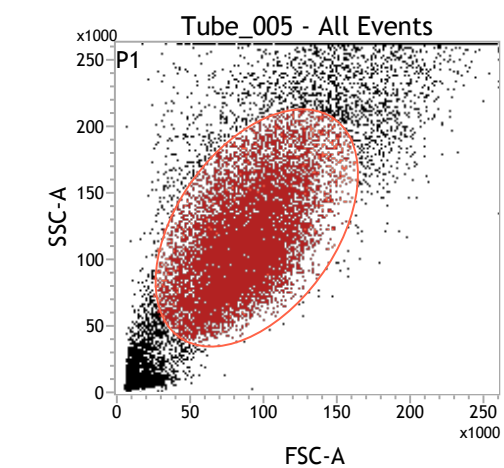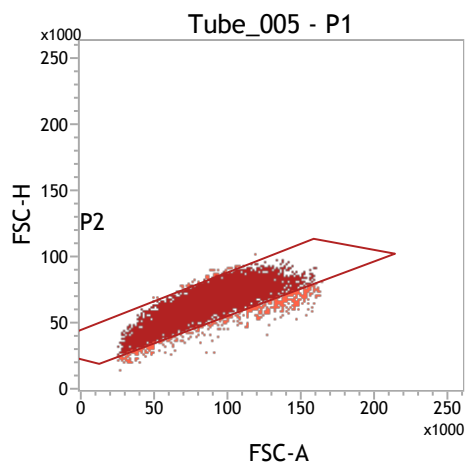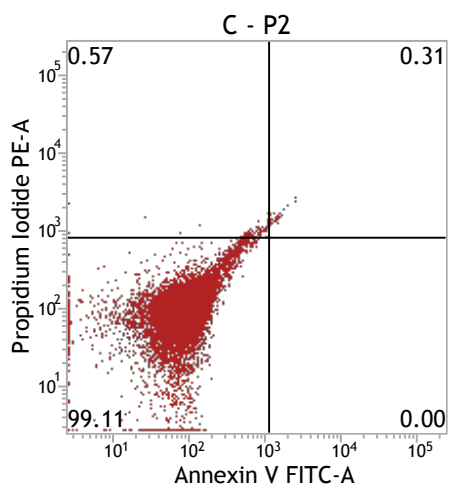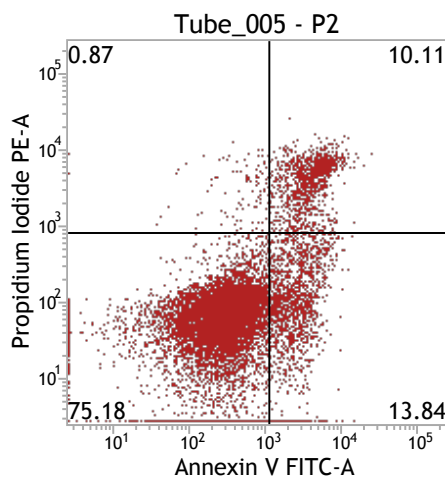

H<sub>2</sub>O<sub>2</sub>

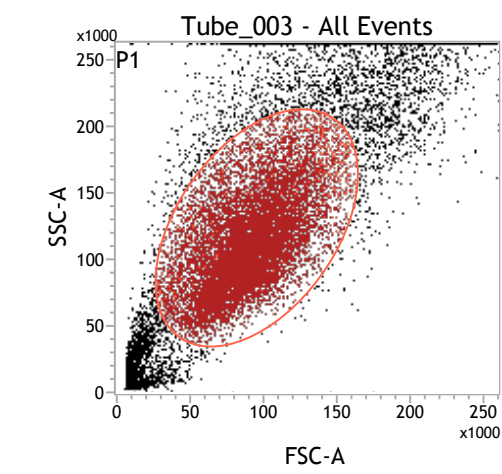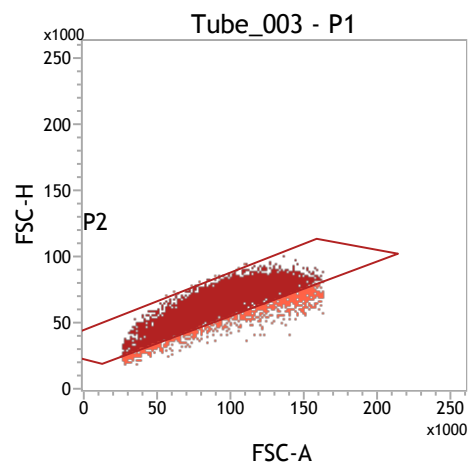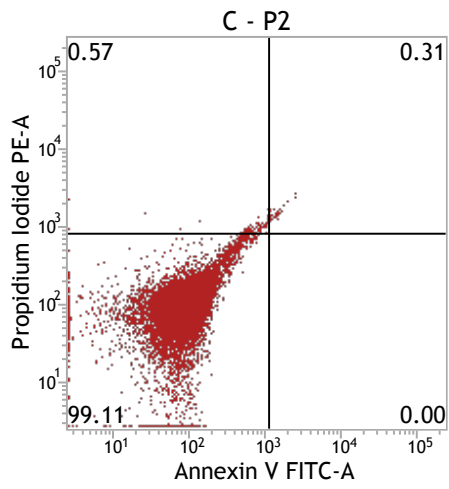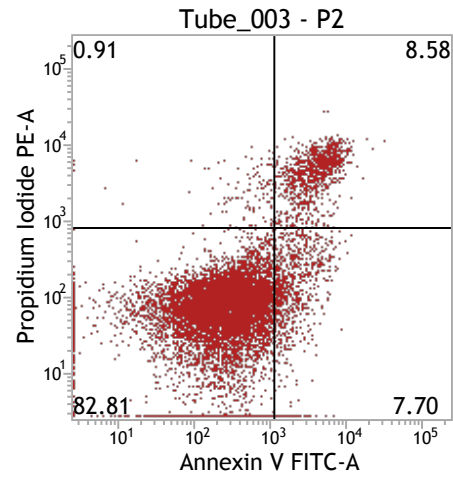

H<sub>2</sub>O<sub>2</sub>+Resveratrol

Supplement: Supplementary file 2 [file Data_Sheet_2.PDF]

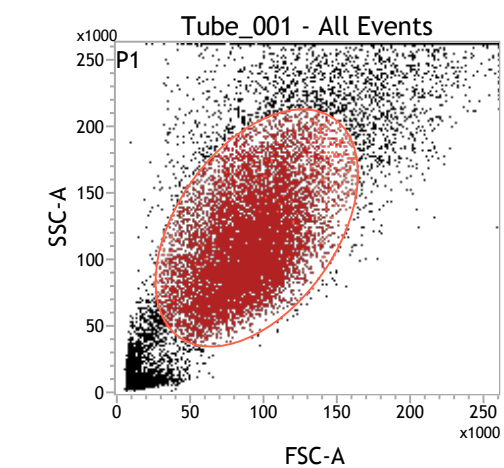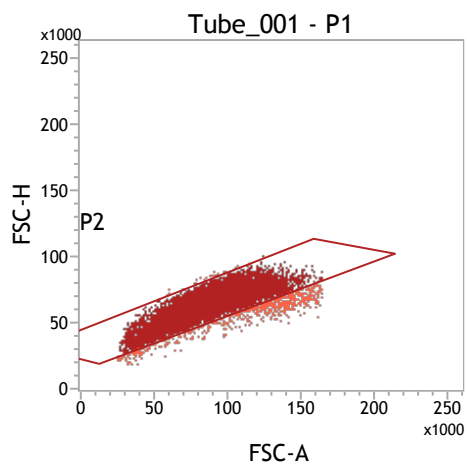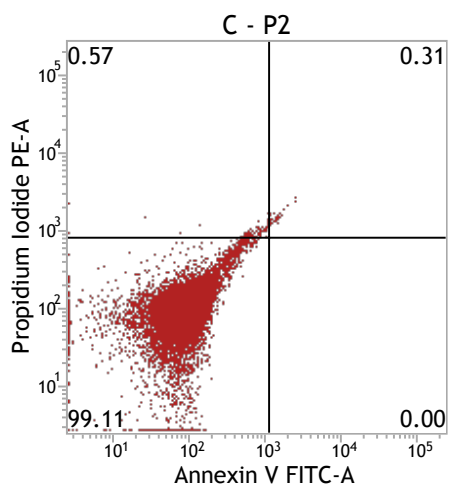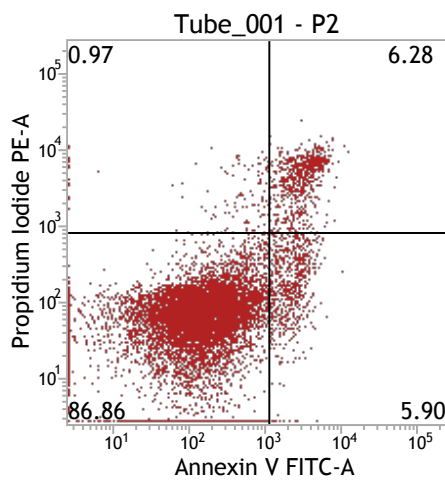

Blank

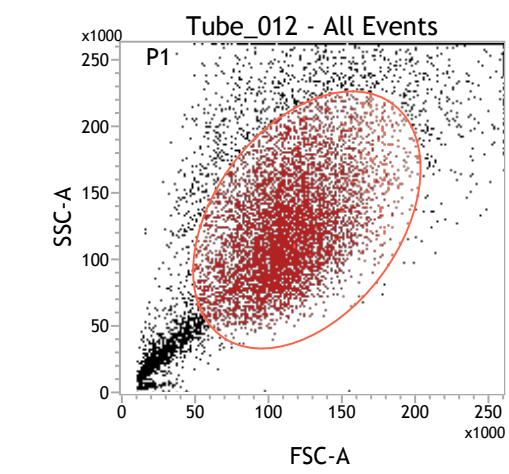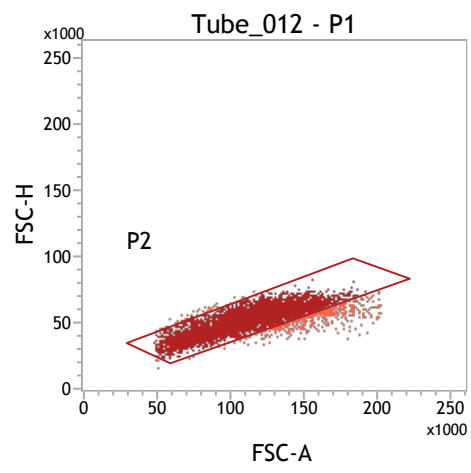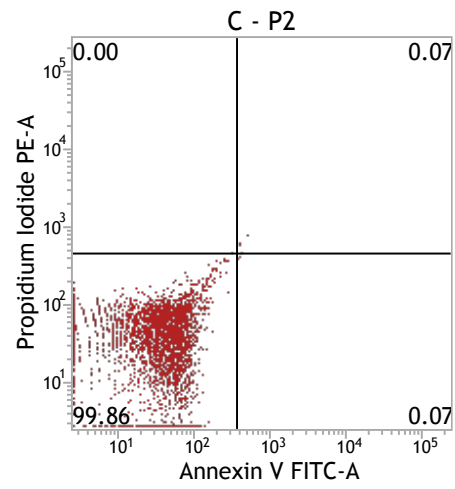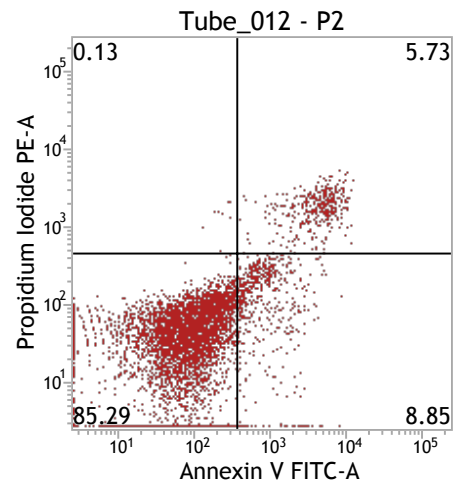

SI -NC

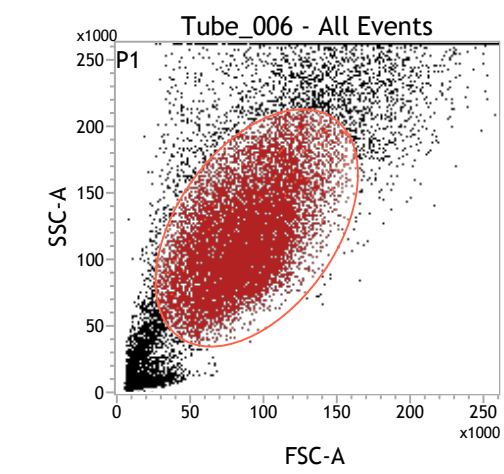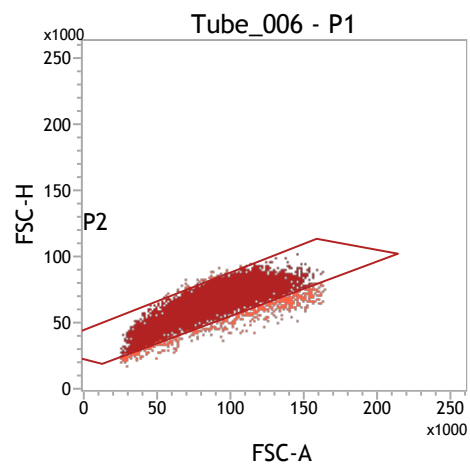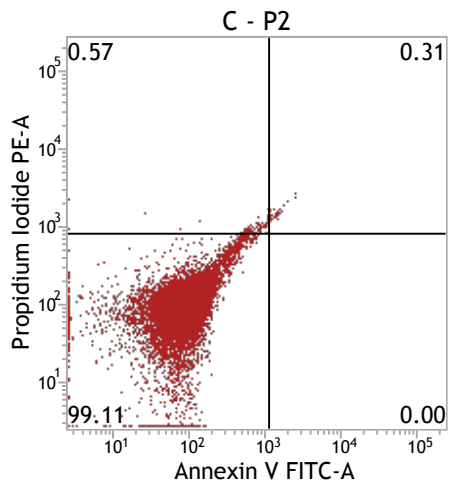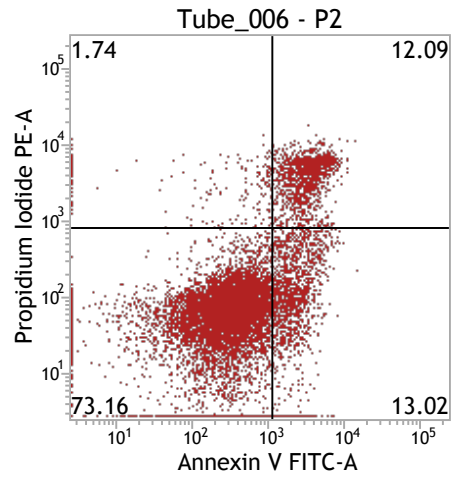

SI-MEF2A

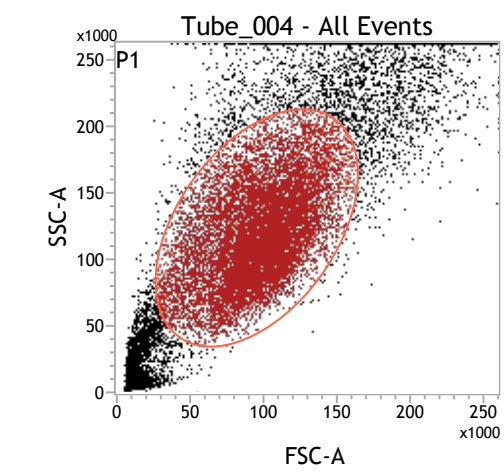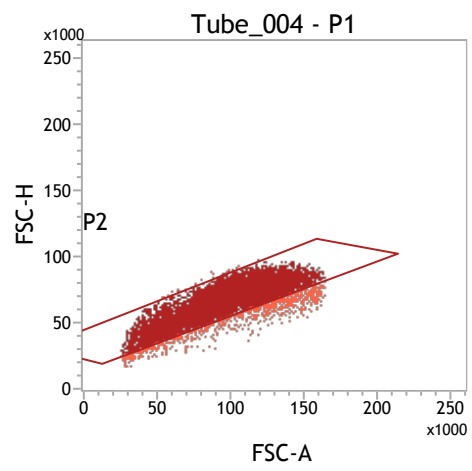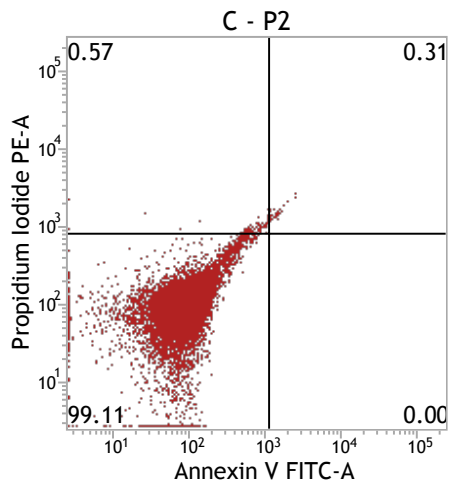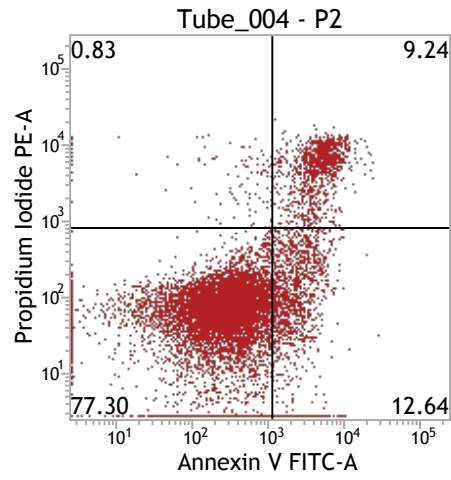

SI-MEF2A + Resveratrol

Supplement: Supplementary file 3 [file Data_Sheet_3.PDF]

Figure 1C

Western blot

1 st


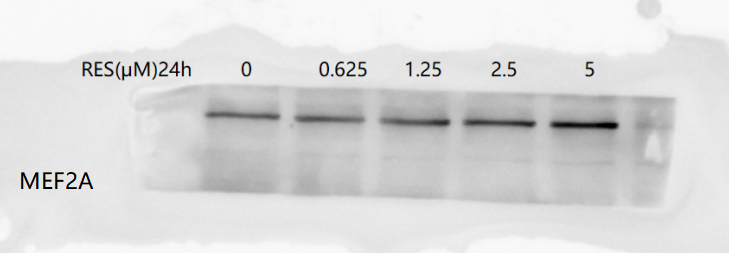


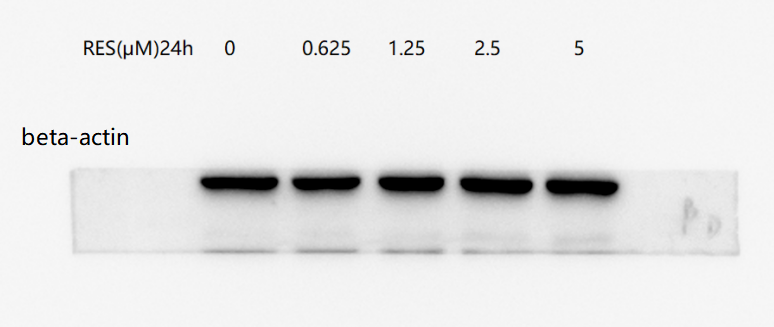


2nd


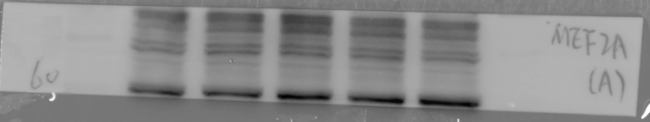


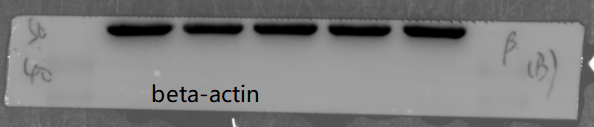


3rd


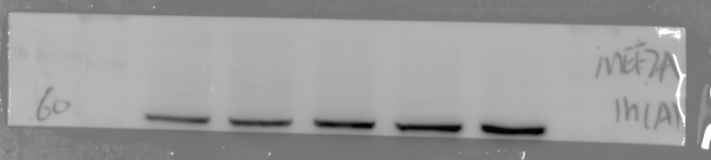


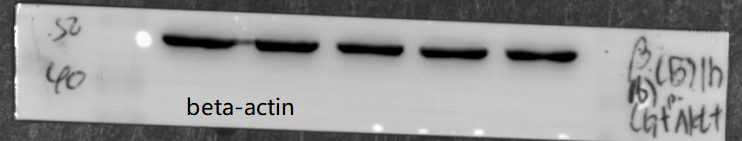

Supplement: Supplementary file 6 [file Table_3.DOCX]

Figure 2B western blot

1 st


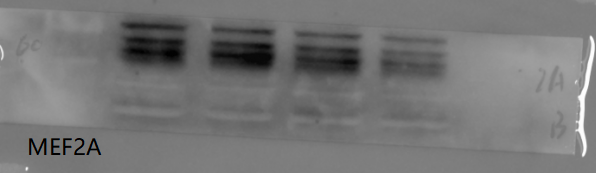


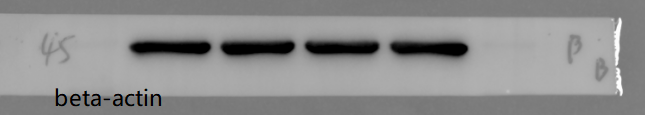


2 nd


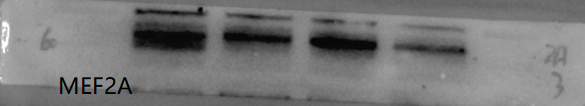


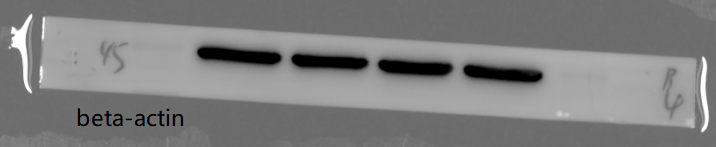


3 rd


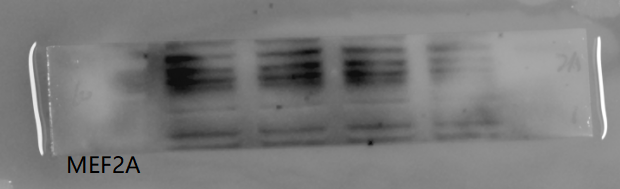


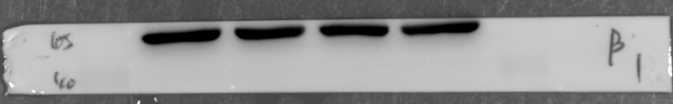


Figure 2C western blot

1 st


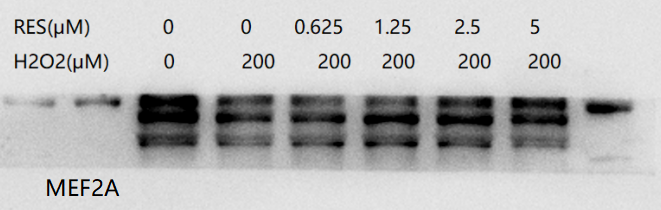


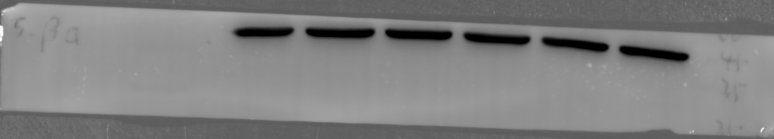


2 nd


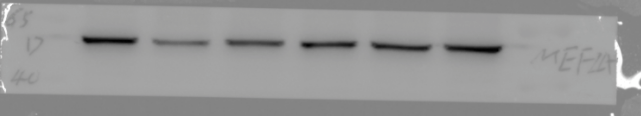


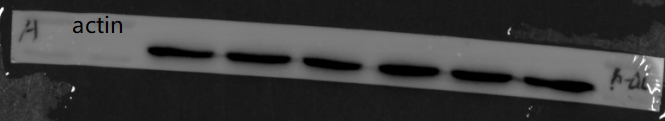


3 rd


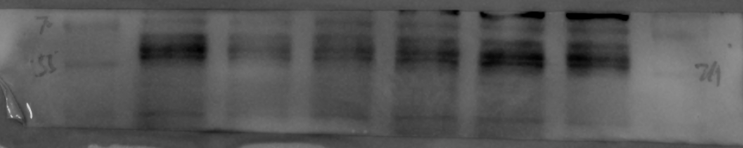


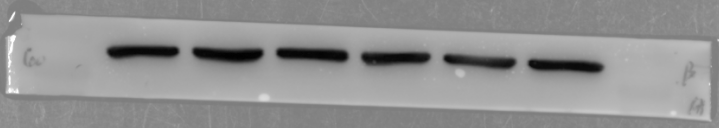

Supplement: Supplementary file 8 [file Table_5.DOCX]

Figure 6A WB

1 st


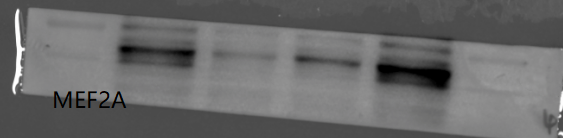


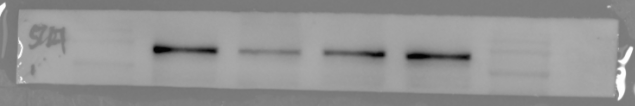


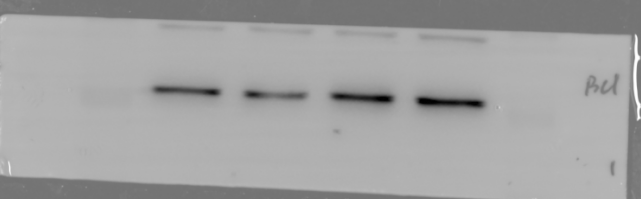


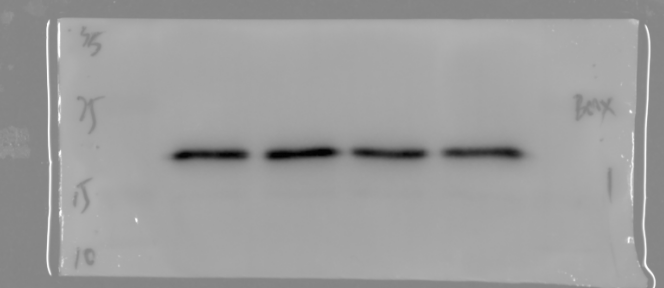


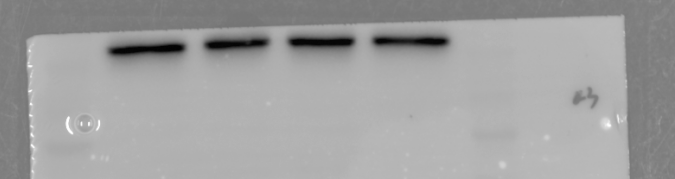


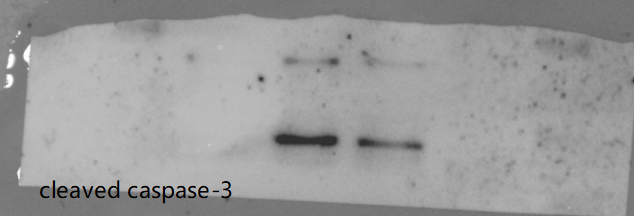


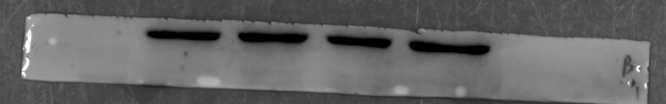


2 nd


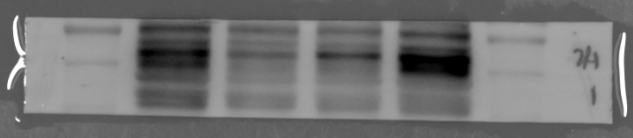


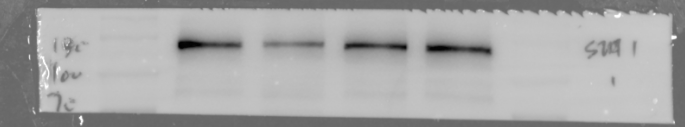


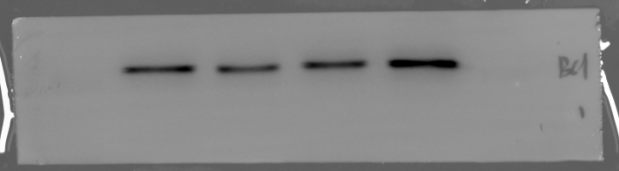


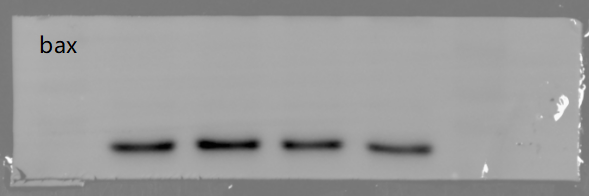


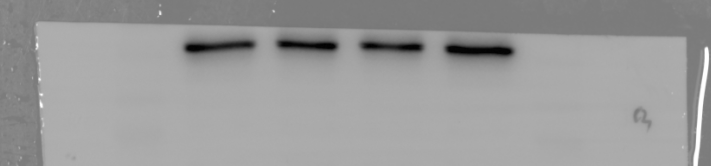


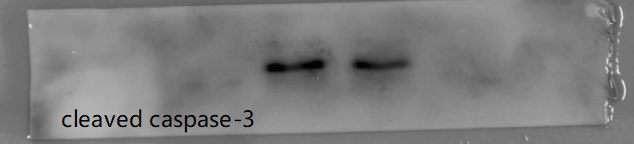


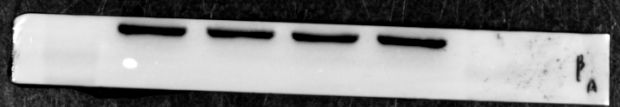


3 nd


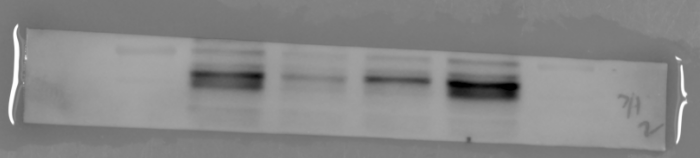


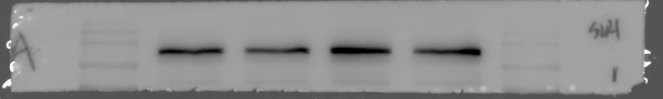


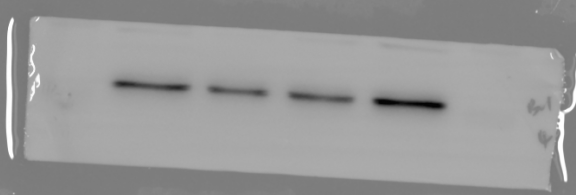


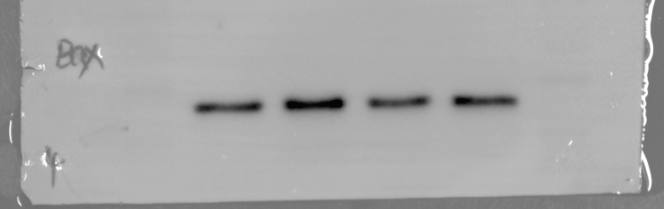


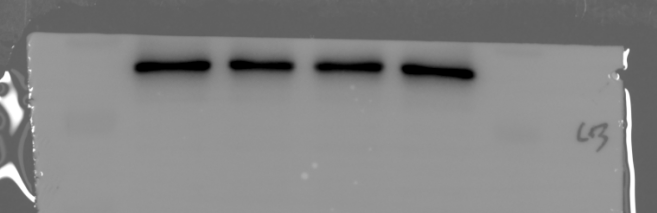


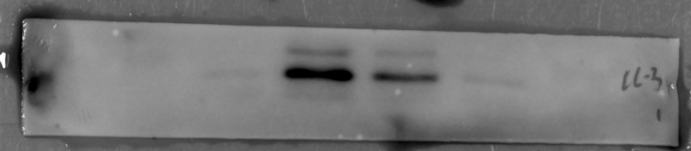


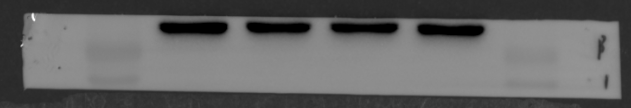


Figure 6B wb

1 st


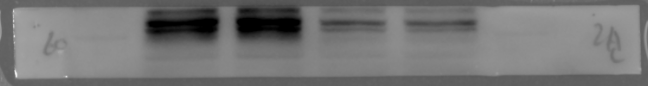


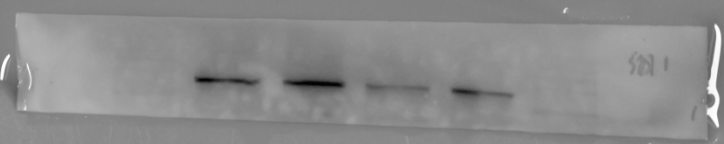


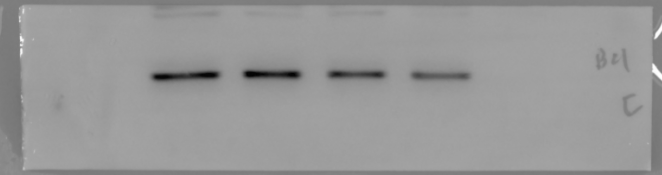


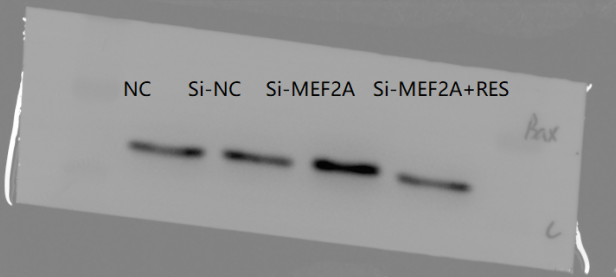


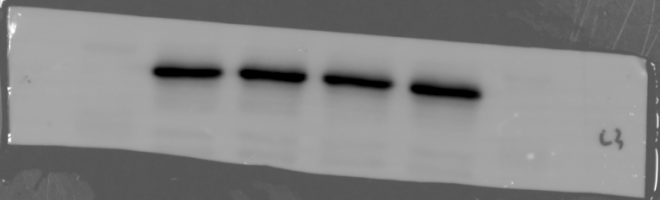


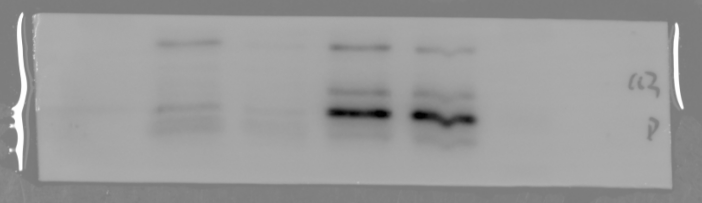


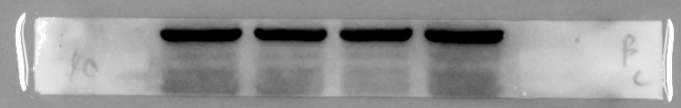


2 nd


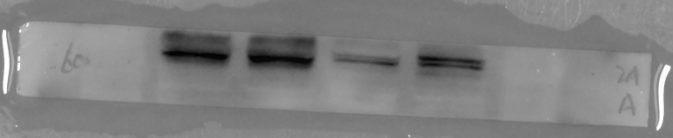


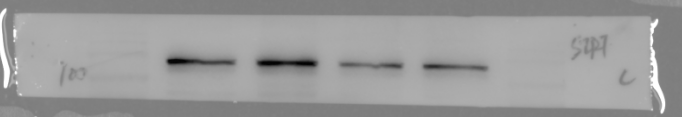


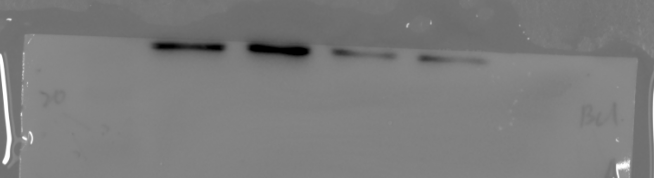


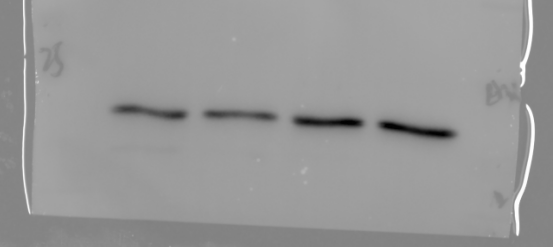


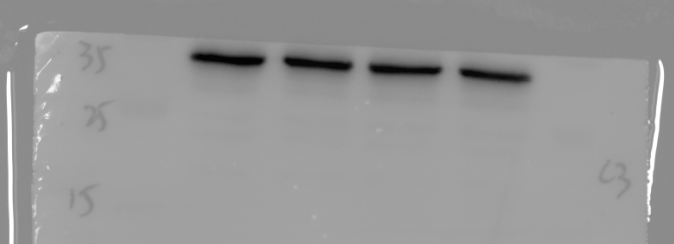


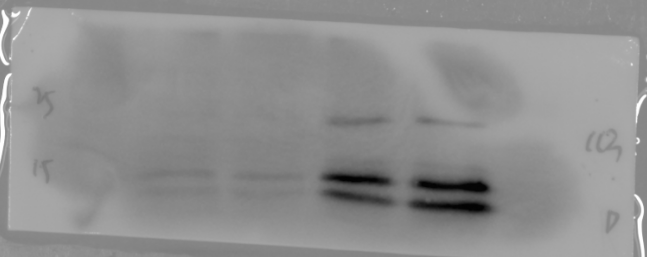


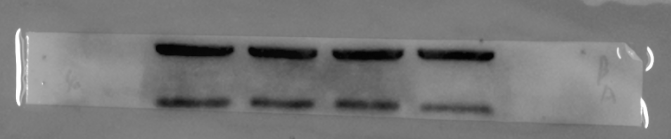


3 rd


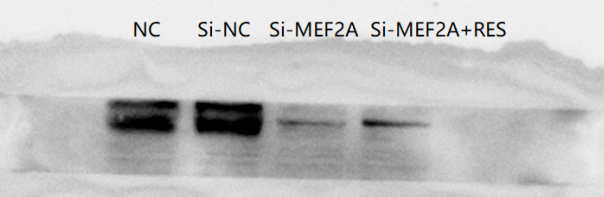


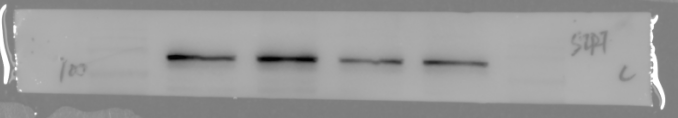


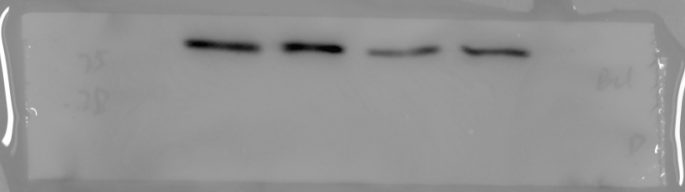


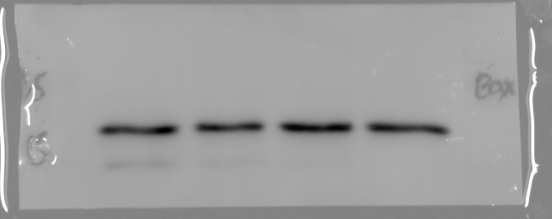


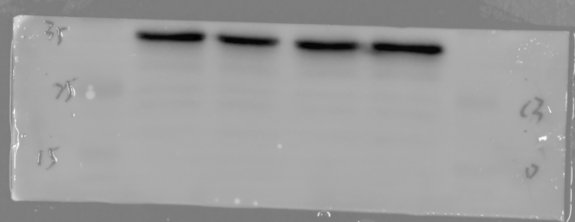


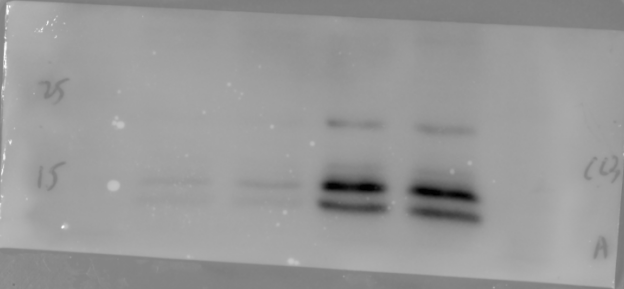


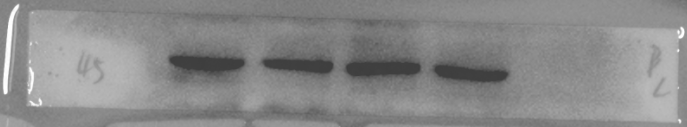

Supplement: Supplementary file 10 [file Table_7.DOCX]

Figure 7A western blot

1 st

Wild type si-NC AAV1 si-MEF2A AAV1


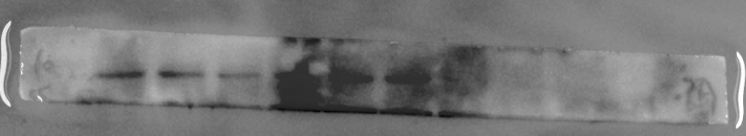


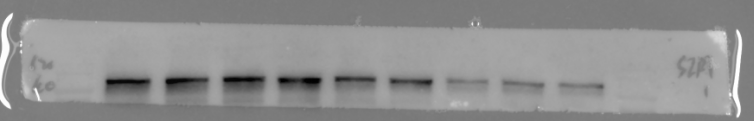


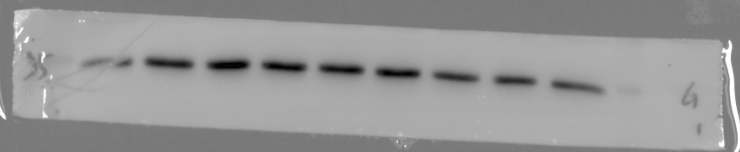


Repeated WB test


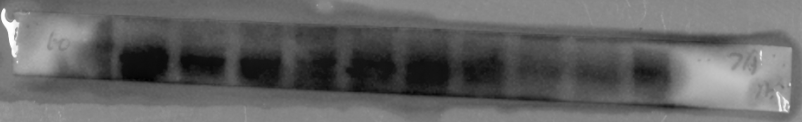


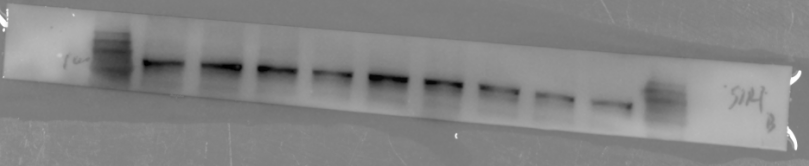


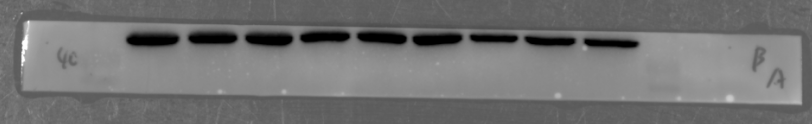

Supplement: Supplementary file 11 [file Table_8.DOCX]
